# Supplementary material for: What are the risks and benefits of temporarily discontinuing medications to prevent acute kidney injury? A systematic review and meta-analysis
Source: BMJ Open. 2017 Apr 7;7(4):e012674. doi: 10.1136/bmjopen-2016-012674 (PMC5541442; doi:10.1136/bmjopen-2016-012674)
Supplement: supplementary appendix [file bmjopen-2016-012674supp001.pdf]

Appendix 1: Search Strategy Medline
Language: all
Date parameters: all
Search Strategy:
1 ((sick day$ or well day$) adj2 (management or protocol$ or recommendation$ or rule$)).ti,ab. (40)
2 ((drug$ or pill$ or medicin$ or medication$) adj2 holiday$).ti,ab. (396)
3 1 or 2 (436)
4 exp "Angiotensin Receptor Antagonists"/ (17888)
5 ((angiotensin adj3 (receptor$ adj2 (antagonist$ or blocker$))) or arb or arbs).ti,ab. (11806)
6 (candesartan or amias or eprosartan or teveten or irbesartan or aprovel or coaprovel or losartan or cozaar or cozaar-comp or olmesartan or olmetec or sevikar or telmisartan or micardis or valsartan or diovan or co-diovan).ti,ab. (14149)
7 exp Angiotensin-Converting Enzyme Inhibitors/ (39220)
8 ((ace or acei or ((angiotensin adj converting adj2 enzyme*) or ace or kininase)) adj2 (inhibitor* or antagonist*)).ti,ab. (29275)
9 (captopril or ecopace or kaplon or capoten or co-zidocapt or capto-co or capozide or cilazapril or vascace or enalapril or ednyt or innovace or innozide or fosinopril or imidapril or tanatril or lisinopril or zestril or carace or zestoretic or moexipril or perdix or perindopril or coversyl or quinapril or quinil or accupro or accuretic or ramipril or tritace or triapin or trandolapril or gopten or tarka).ti,ab. (22409)
10 (renin adj4 (antagonist$ or blocker$ or inhibitor$)).ti,ab. (3552)
11 aliskiren.ti,ab. (868)
12 exp Diuretics/ (71825)
13 (diuretic$ or thiazide$ or indapamide or chlortalidone or bedroflumethiazide or xipamide or metaolozone or cyclopenthiazide or furosemide or bumetanide or torasemide or amiloride or triamterene or spironalactone or eplerenone or co-amilofruse or co-amilozide or mannitol).ti,ab. (67540)
14 exp Mineralocorticoid Receptor Antagonists/ or aldosterone antagonist$.ti,ab. (8409)
15 exp Anti-Inflammatory Agents, Non-Steroidal/ (161061)
16 (nsaid$ or ibuprofen or naproxen or fenoprofen or ketoprofen or diclofenac or aceclofenac or etodolac or indometacin or mefenamic acid or meloxicam or nabumetone or phenylbutazone or piroxicam or sulindac or tenoxicam or tolenamic acid or etoricoxib or celecoxib or acemetacin or dexibuprofen or dexketoprofen or flurbiprofen or tiaprofenic acid).ti,ab. (50654)
17 aspirin.ti,ab. (38487)
18 Metformin/ (7874)
19 metformin.ti,ab. (10748)
20 exp Sulfonylurea Compounds/ (16449)
21 (sulfonylurea$ or sulphonylurea$ or acetohexamide or carbutamide or chlorpropamide or gliclazide or glipizide or glyburide or tolazamide or tolbutamide or glibenclamide or glimepiride or glibornuride or gliquidone or glisoxepide or glyclopyramide or glimipramide).ti,ab. (20419)
22 or/4-21 (378495)
23 Withholding Treatment/ (9466)
24 (withhold$ or withheld$ or "non use" or withdraw$ or avoid$ or restart$ or continu$ or discontinu$ or stop$ or suspend$ or suspension or ceas$ or cessation).ti,ab. (1375429)
25 or/23-24 (1381746)
26 exp angiotensin ii type 1 receptor blockers/ad, ae, ct, tu, to or angiotensin ii type 2 receptor blockers/ad, ae, tu (6817)
27 exp Angiotensin-Converting Enzyme Inhibitors/ad, ae, ct, tu, to (27160)
28 "Angiotensin Receptor Antagonists"/ad, ae, ct, tu (1189)
29 exp Mineralocorticoid Receptor Antagonists/ad, ae, ct, tu, to (4429)
30 exp Diuretics/ad, ae, ct, tu, th, to or exp Anti-Inflammatory Agents, Non-Steroidal/ad, ae, ct, tu, to or Metformin/ad, ae, ct, tu, to (132402)
31 exp Sulfonylurea Compounds/ad, ae, ct, tu, to (7005)
32 ((ace$ or arb$ or diuretic$ or thiazide$ or nsaid$ or metformin or sulfonylurea$ or sulphonylurea$ or DANS) adj3 (side effect$ or adverse effect$ or adverse event$ or danger$ or injur$ or toxic$ or nephrotoxic$)).ti,ab. (5219)

33 or/26-32 (167054)
34 sepsis/ or exp bacteremia/ or shock, septic/ (81961)
35 (sepsis or septic).ti,ab. (94667)
36 ((toxic or endotoxic) adj shock*).ti,ab. (5911)
37 septic?emi*.ti,ab. (17808)
38 (blood stream adj2 infect*).ti,ab. (794)
39 Diarrhea/ (39641)
40 (diarrhoea* or diarrhea*).ti,ab. (81268)
41 Vomiting/ (19797)
42 (vomit* or emesis).ti,ab. (55475)
43 ((critical or serious or acute or intercurrent or concurrent) adj3 illness$).ti,ab. (20530)
44 Influenza, Human/ or *critical illness/ (47114)
45 influenza.ti,ab. (71591)
46 ((sodium or volume) adj3 depletion).ti,ab. (2605)
47 Dehydration/ (10728)
48 dehydration.ti,ab. (22570)
49 ((urinary or respiratory or skin or viral or bacterial or major or serious) adj4 infection$).ti,ab. (170172)
50 (UTI$ or RTI$).ti,ab. (534252)
51 exp *Surgical Procedures, Operative/ (1498735)
52 (surger$ or surgical or operation or operations or operativ$).ti,ab. (1585555)
53 exp Contrast Media/ (95342)
54 ((contrast$ or radiocontrast$) adj3 (agent$ or material$ or medium or media)).ti,ab. (49004)
55 or/34-54 (3590845)
56 exp Acute Kidney Injury/ (35200)
57 ((acute or early) adj (kidney or renal) adj (failure* or injur* or insufficien* or dysfunction* or impair*)).ti,ab. (31135)
58 (acute adj3 (kidney necrosis or tubul* necrosis)).ti,ab. (2972)
59 AKI.ti,ab. (4409)
60 Kidney Diseases/ci [Chemically Induced] (9718)
61 Renal Insufficiency/ci [Chemically Induced] (1216)
62 or/56-61 (58312)
63 exp *acute kidney injury/pc, ci, co, th (10689)
64 *Kidney Diseases/pc, ci, co (11347)
65 *Renal Insufficiency/pc, ci, co (1991)
66 ((AKI or acute kidney injury or acute renal injury or acute kidney failure or acute renal failure or acute kidney necrosis or acute tubular necrosis or acute kidney tubular necrosis or acute kidney insufficienc$ or acute renal insufficienc$ or acute kidney impair$ or acute renal impair$ or acute kidney dysfunction or acute renal dysfunction) adj3 (adverse event$ or adverse effect$ or mortality or morbidity or death$ or prevent$ or treat or treatment or incidence or caus$ or complication$ or minimiz$)).ti,ab. (6112)
67 or/63-66 (28252)
68 (kidney$ or renal or nephro$).mp. (929261)
69 3 and 68 (20)
70 22 and 25 and 62 (924)
71 22 and 55 and 62 (1468)
72 33 and 67 (2411)
73 69 or 70 or 71 or 72 (3765)
74 letter/ (877999)
75 editorial/ (377501)
76 news/ (168594)
77 exp historical article/ (333817)
78 Anecdotes as topic/ (4624)
79 comment/ (625733)
80 case report/ (1731454)
81 (letter or comment$).ti. (102853)
82 animals/ not humans/ (3943670)

83 exp Animals, Laboratory/ (745711)
84 exp Animal Experimentation/ (6628)
85 exp Models, Animal/ (436993)
86 exp rodentia/ (2737281)
87 (rat or rats or mouse or mice).ti. (1142491)
88 or/74-87 (8050101)
89 73 not 88 (2085)
90 exp child/ or exp infant/ or (child$ or infant$ or neonat$ or newborn$ or baby or babies or p?ediatric).ti. (2239441)
91 exp adult/ or adult$.ti. (5842639)
92 90 not 91 (1539523)
93 89 not 92 (1924)
