# Supplementary material for: What are the risks and benefits of temporarily discontinuing medications to prevent acute kidney injury? A systematic review and meta-analysis
Source: BMJ Open. 2017 Apr 7;7(4):e012674. doi: 10.1136/bmjopen-2016-012674 (PMC5541442; doi:10.1136/bmjopen-2016-012674)
Supplement: Author's manuscript [file bmjopen-2016-012674.draft_revisions.pdf]

BMJ Open

What are the risks and benefits of temporarily discontinuing medications to prevent acute kidney injury? A Systematic Review

Journal:	*BMJ Open*
Manuscript ID	bmjopen-2016-012674
Article Type:	Research
Date Submitted by the Author:	16-May-2016
Complete List of Authors:	Whiting, Penny; The National Institute for Health Research Collaboration for Leadership in Applied Health Research and Care West (NIHR CLAHRC West) at University Hospitals Bristol NHS Foundation Trust; University of Bristol, School of Social and Community Medicine<br>Morden, Andrew; The National Institute for Health Research Collaboration for Leadership in Applied Health Research and Care West (NIHR CLAHRC West) at University Hospitals Bristol NHS Foundation Trust; University of Bristol, School of Social and Community Medicine<br>Tomlinson, Laurie; UK Renal Registry; London School of Hygiene and Tropical Medicine<br>Caskey, Fergus; University of Bristol; UK Renal Registry<br>Blakeman, Thomas; University of Manchester, School of Community Based Medicine; National Institute for Health Research Collaboration for Leadership in Applied Health Research and Care (NIHR CLAHRC) Greater Manchester, Centre for Primary Care, Institute of Population Health<br>Tomson, Charles; Freeman Hospital, Department of Renal Medicine<br>Stone, Tracey; The National Institute for Health Research Collaboration for Leadership in Applied Health Research and Care West (NIHR CLAHRC West) at University Hospitals Bristol NHS Foundation Trust; University of Bristol, School of Social and Community Medicine<br>Richards, Alison; The National Institute for Health Research Collaboration for Leadership in Applied Health Research and Care West (NIHR CLAHRC West) at University Hospitals Bristol NHS Foundation Trust; University of Bristol, School of Social and Community Medicine<br>Savovic, Jelena; University of Bristol, NIHR CLAHRC West; University of Bristol, School of Social and Community Medicine<br>Horwood, Jeremy; The National Institute for Health Research Collaboration for Leadership in Applied Health Research and Care West (NIHR CLAHRC West) at University Hospitals Bristol NHS Foundation Trust; University of Bristol, UK, School of Social and Community Medicine
**Primary Subject Heading**:	Renal medicine
Secondary Subject Heading:	Evidence based practice
Keywords:	Acute kidney injury, Medication discontinuation, Sick day rules, Angiotensin-converting enzyme inhibitors, Angiotensin receptor blockers, NSAIDs

For peer review only

What are the risks and benefits of temporarily discontinuing medications to prevent acute kidney injury? A Systematic Review

Penny Whiting[1,2,], Andrew Morden[1,2*], Laurie A Tomlinson[3,4], Fergus Caskey[2,3], Thomas Blakeman[5,6], Charles Tomson[7], Tracey Stone[1,2], Alison Richards[1,2], Jelena Savović[1,2], Jeremy Horwood[1,2]

* Corresponding Author: penny.whiting@bristol.ac.uk
University Hospitals Bristol NHS Foundation Trust, 9th Floor, Whitefriars, Lewins Mead, Bristol BS1 2NT, Tel. +44 117 34 212 73

[1]The National Institute for Health Research Collaboration for Leadership in Applied Health Research and Care West (NIHR CLAHRC West) at University Hospitals Bristol NHS Foundation Trust, Bristol, UK

[2]School of Social and Community Medicine, University of Bristol, Bristol, UK

[3]UK Renal Registry, Bristol, UK

[4]Department of Non-communicable Disease Epidemiology, London School of Hygiene and Tropical Medicine, London, UK

[5]Centre for Primary Care, Institute of Population Health, The University of Manchester, Manchester, UK

[6]National Institute for Health Research Collaboration for Leadership in Applied Health Research and Care (NIHR CLAHRC) Greater Manchester, Centre for Primary Care, Institute of Population Health, University of Manchester, Manchester, UK

[7]Department of Renal Medicine, Freeman Hospital, Newcastle Upon Tyne Hospitals Foundation Trust, Tyne and Wear, UK

Keywords:

Acute kidney injury, Medication discontinuation, Sick day rules, Angiotensin-converting enzyme inhibitors, Angiotensin receptor blockers, NSAIDs

Word count: Article: 3483; Abstract: 295; Figures: 4; Table: 3

For peer review only - http://bmjopen.bmj.com/site/about/guidelines.xhtml

Abstract

Objectives: To summarise evidence on temporary discontinuation of medications to prevent acute kidney injury (AKI).

Design: Systematic review and meta-analysis of randomized and non-randomized studies.

Participants: Adults taking diuretics, angiotensin-converting-enzyme inhibitors, angiotensin receptor blockers, direct renin inhibitors, non-steroidal anti-inflammatories, metformin or sulfonylureas, experiencing inter-current illnesses, radiological or surgical procedures.

Interventions: Temporary discontinuation of any of the medications of interest.

Primary and secondary outcome measures: Risk of AKI. Secondary outcome measures were eGFR and creatinine at 24 hours, urea, systolic and diastolic blood pressure, death, clinical outcomes and biomarkers.

Results: Six studies were included (1,663 participants), three randomised trials and three prospective cohort studies. Mean age ranged from 65 to 73 years, the proportion of women ranged from 31 to 52%. All studies were in hospital settings; five evaluated discontinuation of medication prior to coronary angiography and one prior to cardiac surgery. Five studies evaluated discontinuation of ACE inhibitors and ARBs, one small cohort study looked at discontinuation of NSAIDS. No studies evaluated discontinuation of medication in the community following an acute inter-current illness. There was an increased risk of AKI of around 15% in those in whom medication was continued compared to those in whom it was discontinued (RR 1.17, 95% CI 0.99, 1.38; 5 studies). When only results from RCTs were pooled, the increase in risk was almost 50% (RR 1.48, 95% CI 0.84, 2.60; 3 RCTs) but the confidence interval was wider. There was no difference between groups for any secondary outcomes.

Conclusions: There is low quality evidence that withdrawal of ACE inhibitors/ARBs prior to coronary angiography and cardiac surgery may reduce the incidence of AKI. There is no evidence of the impact of drug cessation interventions on AKI incidence during inter-current illness in primary or secondary care.

For peer review only - http://bmjopen.bmj.com/site/about/guidelines.xhtml

Systematic review registration: PROSPERO CRD42015023210

Article summary

Strengths and limitations of this study:

- We have conducted a thorough systematic review of the evidence from studies that have examined interventions involving temporary discontinuation of medications to prevent or minimise the severity, or consequences, of AKI.
- This is a topic of major importance due to interventions currently being implemented to reduce the risk of AKI throughout the UK and internationally
- Broad eligibility criteria included both randomized and non-randomized studies; Primary and secondary care; inter-current illness or a radiological/surgical procedure; planned and unplanned settings.
- The strength of the conclusion is limited by the quality and number of studies, and absence of evidence for important settings and classes of medications.

For peer review only
For peer review only - http://bmjopen.bmj.com/site/about/guidelines.xhtml

Background

Acute kidney injury (AKI) is a sudden decline in renal function, affecting up to 20% of people admitted to hospital, and is strongly associated with increased mortality and longer duration of hospital stay.[1] Historically recognition and treatment of AKI has been poor.[2] Recent comprehensive initiatives in the UK have focussed on improving awareness and treatment of people with or at risk of AKI.[3] It is thought that a substantial proportion of AKI is triggered or exacerbated by prescribed medications, particularly during times of physiological stress such as inter-current illness, surgery or radiocontrast imaging. [4] These medications include angiotensin-converting-enzyme inhibitors (ACEI), Angiotensin Receptor Blockers (ARB), diuretics, non-steroidal anti-inflammatory drugs (NSAIDs). Under the same circumstances reduced excretion of metformin is associated with an increased risk of lactic acidosis while sulfonylureas can lead to a greater incidence of hypoglycaemia. Therefore, many clinicians, expert consensus statements and guidelines recommend that some or all of these medications are stopped prior to elective or emergency procedures, or when patients become unwell with symptoms of severe infection. [5 6] Initiatives advising patients prescribed these medications to temporarily stop taking them when they become unwell (so called 'sick-day rules') have been implemented throughout Scotland and in local initiatives across the UK. [7] However, the evidence base to support these recommendations is unclear, and the overall benefit remains controversial. [8]

We conducted a systematic review and meta-analysis of the randomised and non-randomized studies that have examined temporary discontinuation of all or any of these medications in patients in primary or secondary care at risk of AKI or with newly diagnosed AKI as a result of an inter-current illness or a radiological/surgical procedure (planned or unplanned).

For peer review only - http://bmjopen.bmj.com/site/about/guidelines.xhtml

Methods

Systematic review methods followed guidance from the Centre for Reviews and Dissemination (CRD) [9] and the Cochrane Collaboration; [10] this review is reported according to the PRISMA guidelines. [11] The review followed a predefined published protocol. [12]

Study Eligibility criteria

Studies, both randomized and non-randomized, that evaluated adults (age ≥18 years) who were taking a specified medication and experiencing an inter-current illness or undergoing a radiological/surgical procedure (planned or unplanned) in whom the medication was temporarily discontinued for any reason were eligible for inclusion. Medications of interest were diuretics, ACEIs, ARBs, direct renin inhibitors, NSAIDs, metformin or sulfonylureas. Studies had to report a measure of kidney function (e.g. incidence of AKI, eGFR, or serum creatinine) and include a comparator group consisting of placebo, no treatment or usual care.

Identification and selection of studies

The following databases were searched from inception to January 2016: Embase, Medline, PsycINFO, BIOSIS Citation Index (Web of Science), CINAHL (Cumulative Index to Nursing and Allied Health Literature), Science Citation Index (SCI) (Web of Science), and the Cochrane Central Register of Controlled Trials (CENTRAL). Supplementary searches were undertaken to identify grey literature, completed and ongoing trials, in the following resources: NIH ClinicalTrials.gov (http://www.clinicaltrials.gov), metaRegister of Controlled Trials (http://www.controlled-trials.com), WHO International Clinical Trials Registry Platform (ICTRP) (http://www.who.int/ictrp/en), relevant guidelines (e.g. NICE in the UK) regarding management of AKI. Reference lists of included studies were screened. Details of the Medline search strategy is available as a supplementary appendix.

For peer review only - http://bmjopen.bmj.com/site/about/guidelines.xhtml

For peer review only

Search results and full-text articles were independently assessed for inclusion by two reviewers; disagreements were resolved through consensus or referral to a third reviewer where necessary.

Data extraction and assessment of risk of bias

We extracted data on baseline characteristics (number of participants, participant characteristics, study settings, study design, country, inclusion and exclusion criteria), intervention/exposure related to stopping medication, and outcomes (incidence of AKI, urinary biomarkers, clinical outcomes, creatinine, eGFR, urea and blood pressure). For dichotomous data (for example incidence of AKI) we extracted the number of events and participants in each treatment group and calculated the relative risk (RR) and 95% confidence interval (CI). For continuous data, we extracted the mean and standard deviation in each treatment group and calculated mean differences (MD) and 95% CIs. RCTs were assessed for methodological quality using a draft version of the new Cochrane risk of bias tool[13] that includes items covering allocation bias (random sequence generation, allocation concealment and baseline imbalance), departures from interventions (participant and study personnel blinding, deviations from intended interventions and analysis in groups to which they were randomized), attrition bias (incomplete outcome data and robustness of results to missing data) detection bias (blinding of outcome assessors and likelihood of blinding to have influenced results), and reporting bias (selective reporting of outcome domain being assessed). The ROBINS-I tool was used to assess the risk of bias in non-randomized studies.[14] It includes domains covering bias due to confounding, bias in the selection of participants into the study, bias due to departures from intended interventions, bias due to missing data, bias in taking measurements and bias in the selection of the reported result.

For peer review only - http://bmjopen.bmj.com/site/about/guidelines.xhtml

Data was extracted by one reviewer using a standard data extraction form designed for this review, and checked by a second reviewer. Risk of bias assessment was performed independently by two reviewers. Any disagreements were resolved by consensus or referral to a third reviewer.

Data synthesis

We grouped studies by design (randomized versus non-randomized), population (coronary angiography versus surgery) and outcome. If there were two or more studies assessing the same outcome, data were plotted on a forest plot. If data were considered statistically and clinically sufficiently homogeneous then summary estimates were produced using random effects meta-analysis. For dichotomous outcomes we estimated summary RRs and 95% CIs, for continuous data we estimated summary MDs and 95% CIs. Heterogeneity was investigated using forest plots and the I^2 statistic. Where data were considered too heterogeneous to pool, a narrative synthesis was provided. We used GRADE to rate the overall quality of the evidence for risk of bias, publication bias, imprecision, inconsistency, indirectness, and magnitude of effect.[15]

Results

The searches identified 4316 hits (records) of which 42 were considered potentially relevant and obtained for full text review. A total of 6 studies (1663 participants) were included in the review: three RCTs (522 participants)[16-18] and three prospective cohort studies (1141 participants).[19-21] One study was available only as a conference abstract and so limited details were available for this study.[21]

All studies were conducted in hospital settings: five evaluated discontinuation of medication prior to coronary angiography and one prior to cardiac surgery.[19] All but one study[16] restricted inclusion to

For peer review only - http://bmjopen.bmj.com/site/about/guidelines.xhtml

patients deemed at higher risk of AKI such as those with chronic kidney disease (3 studies),[17 18 20] diabetes (1 study),[21] or a set of criteria that defined patients at high risk (1 study).[19] The most commonly reported co-morbidities included diabetes, hypertension and congestive heart failure. No studies of discontinuation of medication in the community following an acute inter-current illness were found. Studies were conducted in North America, Turkey and Israel. Mean age, where reported, ranged from 65 to 73 years and the proportion of women in the studies ranged from 31 to 52%. Five studies evaluated discontinuation of ACE inhibitors and ARBs, one small cohort study looked at discontinuation of NSAIDs.[20] The time point at which the medication was stopped varied between studies. Three studies reported that medication was stopped 24 hours prior to the procedure,[16 18 21] two (including the one study of surgery) that it was stopped on the morning of the procedure,[17 19] and one (the study of NSAIDs)[20] did not provide details on when medication was stopped. The time point at which medication was started again also varied. One study stated that medication was started up to 96 hours post-procedure[18], one RCT[16] included two intervention arms that compared restarting immediately post-procedure with restarting 24 hours after the procedure and four studies did not report on this. No studies were found that assessed discontinuation of diuretics, metformin or sulfonylureas. Table 1 provides an overview of included studies.

The risk of bias assessment was performed for the primary outcome of incidence of AKI. Two RCTs were judged to have 'some concerns' regarding risk of bias[16 17] and one was rated 'low risk of bias'.[18] The two judged at 'some concern' both had issues with the randomization process; all other bias domains were rated 'low risk'. One provided no information on the methods used to allocate participants to the two treatment groups, the other randomized patients by means of a coin toss but did not provide any information on whether allocation was concealed. Both studies provided a reasonable overview of baseline characteristics, including similarities in timings of baseline kidney function, which suggested that any differences between groups were compatable with chance. The risk of bias assessment highlighted that none of the studies provided information on blinding of

For peer review only - http://bmjopen.bmj.com/site/about/guidelines.xhtml

participants, study personnel or outcome assessors. However, there do not appear to have been any departures from the intended interventions thus knowledge of the assigned intervention appears unlikely to have influenced the study result. The outcome measure was considered relatively objective and therefore also unlikely to have been influenced by knowledge of treatment assignment.

It was not possible to conduct a risk of bias assessment for one of the non-randomized studies[20] as this study did not provide any numerical data and the risk of bias assessment is performed at the result level. One of the non-randomized studies was judged at moderate risk of bias,[19] the other at critical risk of bias.[21] The study judged at critical risk of bias only presented crude outcome data with no adjustment for potential confounding factors. It was judged at low risk of bias for all other domains with the exception of measurement of interventions which was judged at moderate risk of bias as it was not clear exactly how exposure to ACE inhibitors and ARBs was measured. The study judged at moderate risk of bias was judged to have appropriately controlled for confounding factors but the guidance for the ROBINS-I tool states that this domain can only be rated as low risk of bias if the study is considered comparable to a well-perfomed randomized trial.

One study did not provide any numerical data on the effect of discontinuation of medication on patient outcomes.[20] This cohort study, which assessed discontinuation of NSAIDs, only found three patients out of 44 NSAID users who were advised to discontinue their medication prior to coronary angiography. It reported that discontinuation of NSAID was not associated with a lower rate of AKI, but this was limited by the small number of patients in whom medication was discontinued.

All other studies assessed the incidence of AKI (or contrast induced nephropathy) in those in whom medication was stopped prior to the procedure compared to those in whom medication was continued (Figure 2). Three studies defined AKI as an increase in creatinine of 25% or 0.5 mg/dL

For peer review only - http://bmjopen.bmj.com/site/about/guidelines.xhtml

above baseline,[17 18 21] one as an increase in creatinine of 25% above baseline,[16] and one used a slightly different definition of an increase in creatinine of 50% or 0.3mg/dL above baseline.[19] All but one suggested an increased risk of AKI in those in whom medication was continued, but confidence intervals were generally wide. There was an increased risk of AKI of around 15% in those in whom medication was continued compared to those in whom it was discontinued (RR 1.17, 95% CI 0.99, 1.38). Omitting the study judged at critical risk of bias had very little effect on the summary estimate (RR 1.16, 95% CI 0.98, 1.37). When only results from RCTs were pooled, the increase in risk was almost 50% (RR 1.48, 95% CI 0.84, 2.60; 3 RCTs) but the confidence interval was much wider. There was no evidence of heterogeneity for any of these analyses (I^2=0%). Based on GRADE the quality of the evidence was judged as low for the analysis restricted to RCTs and very low when non-randomized studies were included (Table 3). The evidence was downgraded due to imprecision and the likelihood of publication bias for the analysis that included RCTs and for study quality and publication bias for the analysis that included non-randomized trials.

Two studies[16 17] assessed GFR and creatinine at 24 hours post-intervention (Figures 3 and 4). Both suggested no difference in these measures between intervention groups. Other outcomes reported in single studies included urinary biomarkers (structural AKI), clinical endpoints (mortality, myocardial infarction, stroke, congestive heart failure, rehospitalisation, hypertensive treatment) and blood pressure. Generally there was no difference between groups in which medication was stopped and groups in which it was continued for any of these outcomes. Table 2 provides an overview of key outcomes; other outcomes reported in included studies were different ways of measuring these outcomes (e.g. continuous rather than dichotomous data, or change from baseline rather than absolute value).

For peer review only - http://bmjopen.bmj.com/site/about/guidelines.xhtml

Discussion

The results of our meta-analysis demonstrate an approximately 15% increased risk of AKI in those in whom medication was continued compared to those in whom it was discontinued (RR 1.17, 95% CI 0.99, 1.38). When only results from RCTs were pooled, the increase in risk was almost 50% (RR 1.48, 95% CI 0.84, 2.60) but the confidence interval was much wider. Based on the GRADE approach, the quality of the evidence was low when restricted to RCTs and very low when non-randomized studies were included. There was no difference between groups in which medication was stopped and groups in which it was continued for any secondary outcomes but these were mainly assessed in single studies.

This is the first systematic review into a topic of major importance, as interventions of this type are currently being implemented throughout the UK and internationally, with the aim of reducing the incidence and/or severity of AKI. We have used broad inclusion criteria in many databases to capture randomised and non-randomized studies, in primary and secondary care, and for a range of AKI precipitants including inter-current illness and planned or emergency radiological and surgical procedure. However, we have found that the published evidence was sparse and has important limitations. It is focussed in hospital settings, mainly in patients undergoing coronary angiography, restricted to patients who were considered high risk for AKI and predominantly evaluates discontinuation of ACE inhibitors and ARBs. The primary definition of AKI in all studies was based on short-term changes in serum creatinine although definitions varied across studies. The definitions of AKI used in four of the studies may have overestimated the incidence of AKI compared to the currently accepted definition of AKI, which was used in only one study.[19]

Since ACEIs/ARBs reduce glomerular filtration rate but preserve tubular blood flow, a more marked short-term reduction in eGFR may be associated with lower rates of established AKI due to ongoing tubular injury.[22] Indeed, the only study[19] that examined alternate biomarker-based definitions of AKI

For peer review only - http://bmjopen.bmj.com/site/about/guidelines.xhtml

found no effect related to drug cessation. In addition, the longer term impact of AKI in terms of the development of CKD or reductions in baseline GFR was not reported. The reduction in glomerular filtration rate caused by ACEI and ARB treatment is reversible on stopping the drug.[23] This temporary rise in GFR among patients who discontinued the drugs might have masked AKI in the studies included here, given that AKI was defined as a change in serum creatinine from a baseline measurement that was taken prior to drug withdrawal. Recognising the potential for physiological rather than pathological changes in kidney function,[22] future studies will benefit from examining later clinical outcomes including incomplete recovery from AKI (i.e. failure of serum creatinine concentration to return to baseline), chronic kidney disease, and all-cause mortality.

To further quantify the limitations of the studies, we conducted a formal risk of bias assessment using the most recently developed tools. This is the first review to have used both the ROBINS-I tool[14] and the new Cochrane tool for randomised trials. [13] The majority of studies were small and there were some concerns regarding risk of bias in some studies, especially one of the non-randomized studies which was judged at critical risk of bias. Publication bias was not formally assessed in this review because the number of studies was too small for such an assessment to be meaningful. However, our search strategy included a variety of routes to identify unpublished studies and resulted in the inclusion of one conference abstract. Despite this we consider the likelihood of publication bias in this area to be high.

Importantly, there are no studies which evaluate the benefits of stopping medication in the community following acute infection, and no studies that assessed discontinuation of diuretics that could exacerbate AKI, or metformin and/or sulfonylureas, which may accumulate during an episode of AKI. Only one study assessed discontinuation of NSAIDs and only a very small number of patients discontinued these drugs in this study and so it was not possible to draw conclusions regarding the effects of discontinuing NSAIDs.

For peer review only - http://bmjopen.bmj.com/site/about/guidelines.xhtml

At present a number of national organisations provide guidance about medication cessation, as well as many regional schemes and guidelines. The UK NICE guidance published in 2013 recommends consideration of temporarily stopping ACE inhibitors and ARBs in adults having iodinated contrast agents if they have chronic kidney disease with an eGFR less than 40 ml/min/1.73 m^2, and in adults, children and young people with diarrhoea, vomiting or sepsis.[6] In 2015, NHS Scotland and the Scottish Patient Safety Programme initiated a more wide ranging medication cessation intervention. Predominantly via community pharmacists, patients are issued with Sick-Day Rules cards, advising them to stop taking ACEIs/ARBs, NSAIDS, diuretics and metformin when they become unwell with vomiting or diarrhoea, and/or fevers sweats and shaking.[24] Under similar circumstances, guidance from the Canadian Diabetes Association Clinical Practice Guidelines for Chronic Kidney Disease in Diabetes recommends physicians and patients to withhold ACEIs, ARBs, NSAIDs, diuretics, metformin, direct renin inhibitors and Sulfonylureas.[25] This guidance is based on the commonly-held belief that there is an association between the use of ACE Inhibitors/ARBs, diuretics and NSAIDs and the development of AKI, particularly during illness or other physiological insult. The potentially strongest source of evidence, the incidence of AKI in randomised controlled trials of ACEIs and ARBs compared to placebo is poorly described due to variable definitions or absent reporting of kidney related adverse events.[26] A number of observational studies have demonstrated a higher risk of AKI among patients among ACEI/ARB users also taking diuretics and/or NSAIDs compared to those taking ACEIs/ARBs alone,[27-29] or with ACEI/ARB users compared to non-users during acute illness or after surgery.[30 31] As with all observational evidence, these studies carry an inherent risk of associations being due to bias and confounding, particularly confounding by indication, in which patients at higher risk of AKI are more likely to be treated with the drugs of interest making a direct causal effect uncertain.

For peer review only - http://bmjopen.bmj.com/site/about/guidelines.xhtml

Only one of the studies[17] considered in this review was available at the time of development of the NICE guidance for AKI. [6] The guideline development group discuss explicitly the difficulty of issuing guidance regarding medication cessation (for ACEIs/ARBs only) despite limited evidence.[6] They felt that the available evidence for discontinuation was weak but that the “continuing use of ACEIs/ARBs [during acute illness or exposure to iodinated contrast agents] is clearly associated with AKI. In contrast, the temporary suspension of ACEIs/ARBs for a short period seems unlikely to greatly increase the risk of cardiovascular events.” Subsequent evidence regarding the safety of community medication cessation interventions has come from an ongoing evaluation of hospital admissions following introduction of the NHS Scotland scheme, which has shown a stabilisation or fall in hospital admissions with AKI. [24] However, a concurrent fall in heart failure admissions (which might have been expected to increase as a consequence of discontinuation of ACEIs or ARBs amongst patients previously stabilised on these drugs for treatment of heart failure), suggest a secular trend in hospital admissions unrelated to the introduction of the intervention, and interpretation is also limited by the absence of a control population. There remains ongoing disagreement about how the general evidence base should be interpreted to consider the balance of risks and benefits of drug-cessation interventions, particularly during acute illness.[8]

This systematic review includes five additional studies published since the NICE guidance on AKI. Our results show low quality evidence that withdrawal of ACE Inhibitors/ARBs and NSAIDs prior to coronary angiography and cardiac surgery may reduce the incidence of AKI. However, the quality, power and limited scope of these studies reduce the emphasis that can be placed on this finding and have not substantially clarified the evidence base. There is no published evidence of the impact of drug cessation interventions on AKI incidence during inter-current illness in primary or secondary care, of other included medications (NSAIDs, diuretics, sulfonylureas, metformin) or of combinations of medications. We also found no evidence of ongoing studies of interventions on any of these topics.

For peer review only - http://bmjopen.bmj.com/site/about/guidelines.xhtml

The current widespread promotion of 'sick-day guidance' incurs financial and opportunity costs. While the public health impact of sick-day guidance can be evaluated through the novel data flows recently established by NHS England and the UK Renal Registry,[3] more formal controlled evaluation in the form of stepped wedge or cluster randomised trials could be applied to ensure we achieve maximal overall public health benefit.

For peer review only

For peer review only - http://bmjopen.bmj.com/site/about/guidelines.xhtml

Funding

This research is supported by the National Institute for Health Research (NIHR) Collaboration for Leadership in Applied Health Research and Care (CLAHRC) West at University Hospitals Bristol NHS Foundation Trust. Tom Blakeman was partly funded by the NIHR CLAHRC Greater Manchester. The funders had no role in the design of the study, data collection and analysis, decision to publish, or preparation of the manuscript. However, the project outlined in this article may be considered to be affiliated to the work of the NIHR CLAHRC Greater Manchester and NIHR CLAHR West. The views expressed in this article are those of the authors and not necessarily those of the NHS, NIHR or the Department of Health. Laurie Tomlinson is funded by a Wellcome Trust intermediate clinical fellowship (101143/Z/13/Z).

Competing interests

The authors declare that they have no competing interests.

Authors' contributions

CT, TB and FC conceived the idea for the review. PW, AM, JH and LT drafted the article with the support of FC. AR developed the search strategy. FC, LT, TB and CT served as a content experts in the field of AKI. JH served as the overall supervisor and provided input on study methodology. JS provided methodological support. AM, PW and TS undertook screening and data extraction. PW and FC performed the risk of bias assessment. PW and JS performed the GRADE assessment. All authors contributed to the interpretation of results, commented on draft manuscripts and have given their approval for publication.

For peer review only - http://bmjopen.bmj.com/site/about/guidelines.xhtml

Acknowledgements

We would like to thank Dr Tim Jones, NIHR CLAHRC West, for help with assessing the risk of bias in the included studies.

Data sharing

no additional data are available.

For peer review only

For peer review only - http://bmjopen.bmj.com/site/about/guidelines.xhtml

References

1. Chertow GM, Burdick E, Honour M, et al. Acute kidney injury, mortality, length of stay, and costs in hospitalized patients. J Am Soc Nephrol 2005;**16**(11):3365-70.
2. National Confidential Enquiry into Patient Outcome and Death. Acute Kidney Injury: Adding Insult to Injury. 2009. http://www.ncepod.org.uk/2009aki.html.
3. Think Kidneys. Acute Kidney Injury: The NHS campaign to improve the care of people at risk of, or with, acute kidney injury. 2016; (11/4/2016). https://www.thinkkidneys.nhs.uk/aki/.
4. Pannu N, Nadim MK. An overview of drug-induced acute kidney injury. Crit Care Med 2008;**36**(4 Suppl):S216-23.
5. Feehally J, Gilmore I, Barasi S, et al. RCPE UK consensus conference statement: Management of acute kidney injury: the role of fluids, e-alerts and biomarkers. The journal of the Royal College of Physicians of Edinburgh 2013;**43**(1):37-8.
6. National Institute for Health and Clinical Excellence. Acute kidney injury: prevention, detection and management. NICE guideline (CG169), 2013.
7. Scottish Patient Safety Programme. Medicine Sick Day Rules Card. 2015. http://www.scottishpatientsafetyprogramme.scot.nhs.uk/programmes/primary-care/medicine-sick-day-rules-card.
8. Griffith K AC, Blakeman T, Fluck R, Lewington A, Selby N, Tomlinson L, Tomson C. “Sick day rules” in patients at risk of Acute Kidney Injury: an Interim Position Statement from the Think Kidneys Board. 2015. https://www.thinkkidneys.nhs.uk/wp-content/uploads/2015/07/Think-Kidneys-Sick-Day-Rules-160715.pdf.
9. Centre for Reviews and Dissemination. Systematic Reviews: CRD’s guidance for undertaking reviews in health care [Internet]. 2009 http://www.york.ac.uk/inst/crd/SysRev/!SSL!/WebHelp/SysRev3.htm.
10. Higgins JPT, Green S. Cochrane handbook for systematic reviews of interventions [Internet]. 2011 http://www.cochrane-handbook.org/.
11. Moher D, Liberati A, Tetzlaff J, et al. Preferred reporting items for systematic reviews and meta-analyses: the PRISMA statement. Ann Intern Med 2009;**151**(4):264-9, w64.
12. Morden A, Horwood J, Whiting P, et al. The risks and benefits of patients temporarily discontinuing medications in the event of an intercurrent illness: a systematic review protocol. Systematic reviews 2015;**4**:139.
13. Savovic J HJ, Sterne J, Boutron I, Hrobjartsson A. Introducing a revised risk of bias tool for randomized trials. Cochrane Colloquium 2015. http://www.cochranelibrary.com/dotAsset/5bc8d6fd-0604-4bed-beb2-8bc7a74aa4b9.pdf.
14. The ROBINS-I tool (Risk Of Bias In Non-randomized Studies - of Interventions). 2016; (16/5/2016). www.riskofbias.info.
15. Guyatt GH, Oxman AD, Vist GE, et al. GRADE: an emerging consensus on rating quality of evidence and strength of recommendations. BMJ 2008;**336**(7650):924-6.
16. Wolak T, Aliev E, Rogachev B, et al. Renal safety and angiotensin II blockade medications in patients undergoing non-emergent coronary angiography: a randomized controlled study. Isr Med Assoc J 2013;**15**(11):682-7.
17. Rosenstock JL, Bruno R, Kim JK, et al. The effect of withdrawal of ACE inhibitors or angiotensin receptor blockers prior to coronary angiography on the incidence of contrast-induced nephropathy. Int Urol Nephrol 2008;**40**(3):749-55.
18. Bainey KR, Rahim S, Etherington K, et al. Effects of withdrawing vs continuing renin-angiotensin blockers on incidence of acute kidney injury in patients with renal insufficiency undergoing cardiac catheterization: Results from the Angiotensin Converting Enzyme Inhibitor/Angiotensin Receptor Blocker and Contrast Induced Nephropathy in Patients Receiving Cardiac Catheterization (CAPTAIN) trial. Am Heart J 2015;**170**(1):110-6.

For peer review only - http://bmjopen.bmj.com/site/about/guidelines.xhtml

19. Coca SG, Garg AX, Swaminathan M, et al. Preoperative angiotensin-converting enzyme inhibitors and angiotensin receptor blocker use and acute kidney injury in patients undergoing cardiac surgery. Nephrol Dial Transplant 2013;**28**(11):2787-99.
20. Weisbord SD, Mor MK, Resnick AL, et al. Prevention, incidence, and outcomes of contrast-induced acute kidney injury. Arch Intern Med 2008;**168**(12):1325-32.
21. Goksuluk H, Kerimli N, Atmaca Y, et al. Effects of renin-angiotensin-aldosterone system blockers on contrast-induced nephropathy and its association with NGAL levels in diabetic patients undergoing coronary angiography. Eur Heart J 2015;**36**:642.
22. Perazella MA, Coca SG. Three feasible strategies to minimize kidney injury in 'incipient AKI'. Nat Rev Nephrol 2013;**9**(8):484-90.
23. Bakris GL, Weir MR. Angiotensin-converting enzyme inhibitor-associated elevations in serum creatinine: is this a cause for concern? Arch Intern Med 2000;**160**(5):685-93.
24. Morrison C, Wilson M. Medicine sick day rules cards: A safe and effective tool to improve medicines safety in NHS Highland. International Journal of Pharmacy Practice 2015;**23**:92-93.
25. Canadian Diabetes Association. Sick Day Medication List. 2015. http://guidelines.diabetes.ca/browse/appendices/appendix7_2015.
26. Palmer SC, Mavridis D, Navarese E, et al. Comparative efficacy and safety of blood pressure-lowering agents in adults with diabetes and kidney disease: a network meta-analysis. Lancet 2015;**385**(9982):2047-56.
27. Dreischulte T, Morales DR, Bell S, et al. Combined use of nonsteroidal anti-inflammatory drugs with diuretics and/or renin-angiotensin system inhibitors in the community increases the risk of acute kidney injury. Kidney Int 2015;**88**(2):396-403.
28. Lapi F, Azoulay L, Yin H, et al. Concurrent use of diuretics, angiotensin converting enzyme inhibitors, and angiotensin receptor blockers with non-steroidal anti-inflammatory drugs and risk of acute kidney injury: nested case-control study. BMJ 2013;**346**:e8525.
29. Loboz KK, Shenfield GM. Drug combinations and impaired renal function -- the 'triple whammy'. Br J Clin Pharmacol 2005;**59**(2):239-43.
30. Arora P, Rajagopalam S, Ranjan R, et al. Preoperative use of angiotensin-converting enzyme inhibitors/angiotensin receptor blockers is associated with increased risk for acute kidney injury after cardiovascular surgery. Clin J Am Soc Nephrol 2008;**3**(5):1266-73.
31. Plataki M, Kashani K, Cabello-Garza J, et al. Predictors of acute kidney injury in septic shock patients: an observational cohort study. Clin J Am Soc Nephrol 2011;**6**(7):1744-51.

For peer review only

For peer review only - http://bmjopen.bmj.com/site/about/guidelines.xhtml

Figure 1 Flow of studies through the review process

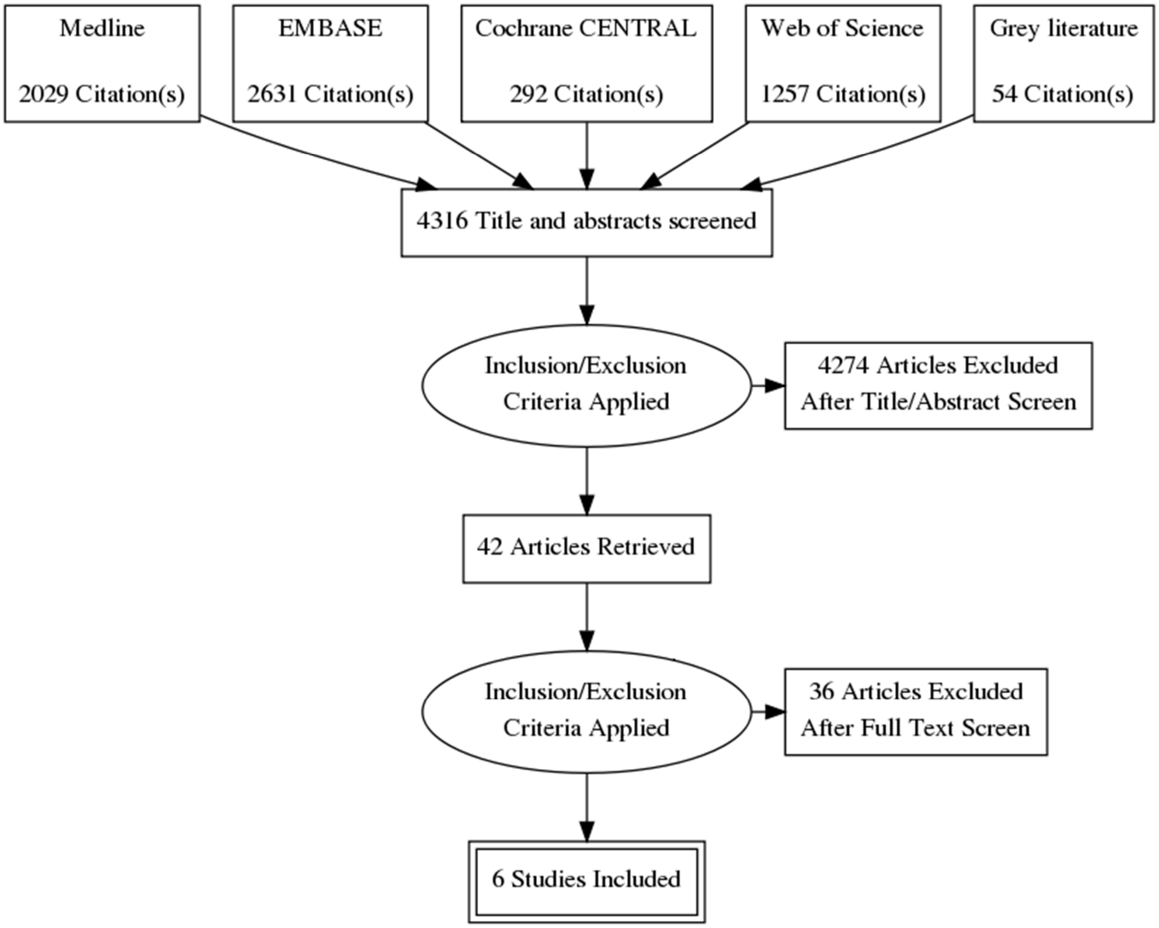

For peer review only - http://bmjopen.bmj.com/site/about/guidelines.xhtml

Figure 2: Forest plot showing risk of AKI in those who stopped medication prior to procedure compared to those who continued medication

Study or Subgroup	continuation Events	continuation Total	discontinuation Events	discontinuation Total	Weight	Risk Ratio IV, Random, 95% CI
**1.1.1 RCT**						
Bainey (2015)	19	102	12	106	6.0%	1.65 [0.84, 3.21]
Rosenstock (2008)	7	113	4	107	1.9%	1.66 [0.50, 5.50]
Wolak (2013) 24h restart	1	33	5	61	0.6%	0.37 [0.05, 3.03]
**Subtotal (95% CI)**		**248**		**274**	**8.5%**	**1.48 [0.84, 2.60]**
Total events	27		21			
Heterogeneity: Tau² = 0.00; Chi² = 1.80, df = 2 (P = 0.41); I² = 0%						
Test for overall effect: Z = 1.37 (P = 0.17)						
**1.1.4 Cohort**						
Coca (2013)	99	231	298	786	89.3%	1.13 [0.95, 1.34]
Goksuluk (2015)	8	41	4	39	2.2%	1.90 [0.62, 5.81]
**Subtotal (95% CI)**		**272**		**825**	**91.5%**	**1.14 [0.96, 1.36]**
Total events	107		302			
Heterogeneity: Tau² = 0.00; Chi² = 0.81, df = 1 (P = 0.37); I² = 0%						
Test for overall effect: Z = 1.54 (P = 0.12)						
**Total (95% CI)**		**520**		**1099**	**100.0%**	**1.17 [0.99, 1.38]**
Total events	134		323			
Heterogeneity: Tau² = 0.00; Chi² = 3.35, df = 4 (P = 0.50); I² = 0%						
Test for overall effect: Z = 1.87 (P = 0.06)						
Test for subgroup differences: Chi² = 0.74, df = 1 (P = 0.39), I² = 0%						

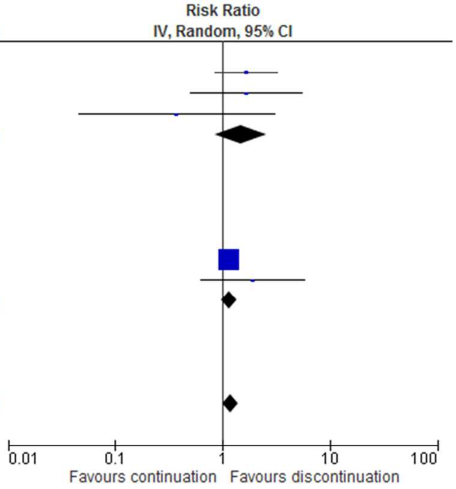

Figure 3: Forest plot showing mean difference in GFR at 24 hours in those who stopped medication prior to procedure compared to those who continued medication

Study or Subgroup	continuation Mean	continuation SD	continuation Total	discontinuation Mean	discontinuation SD	discontinuation Total	Weight	Mean Difference IV, Random, 95% CI
Rosenstock (2008)	48.3	13.9	113	47.2	14.1	107	91.5%	1.10 [-2.60, 4.80]
Wolak (2013) 24h restart	92.99	22.8	33	96.09	26.43	31	8.5%	-3.10 [-15.23, 9.03]
**Total (95% CI)**			**146**			**138**	**100.0%**	**0.74 [-2.80, 4.28]**
Heterogeneity: Tau² = 0.00; Chi² = 0.42, df = 1 (P = 0.52); I² = 0%								
Test for overall effect: Z = 0.41 (P = 0.68)								

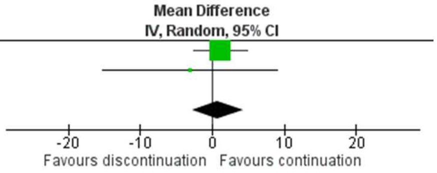

Figure 4: Forest plot showing mean difference in creatinine at 24 hours in those who stopped medication prior to procedure compared to those who continued medication

Study or Subgroup	continuation Mean	continuation SD	continuation Total	discontinuation Mean	discontinuation SD	discontinuation Total	Weight	Mean Difference IV, Random, 95% CI
Rosenstock (2008)	1.5	0.4	113	1.5	0.4	107	55.3%	0.00 [-0.11, 0.11]
Wolak (2013) 24h restart	0.82	0.25	33	0.87	0.23	31	44.7%	-0.05 [-0.17, 0.07]
**Total (95% CI)**			**146**			**138**	**100.0%**	**-0.02 [-0.10, 0.06]**
Heterogeneity: Tau² = 0.00; Chi² = 0.38, df = 1 (P = 0.54); I² = 0%								
Test for overall effect: Z = 0.56 (P = 0.58)								

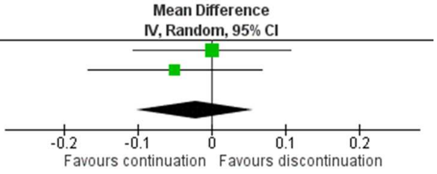

For peer review only - http://bmjopen.bmj.com/site/about/guidelines.xhtml

Table 1: Details of studies included in the review

	Bainey et al (2015)[18]	Rosenstock (2008)[17]	Wolak (2013)[16]	Coca (2013)[19]	Goksuluk (2015)[21]*	Weisbord (2008)[20]
**Study design**	RCT	RCT	RCT	Prospective cohort	Prospective cohort	Prospective cohort
**Sample size**	208	220	94	1017	80	44
**Country**	Canada	United States	Israel	North America	Turkey	United States
**Population**	Coronary angiography	Coronary angiography	Coronary angiography	Cardiac surgery	Coronary angiography	Coronary angiography
**Risk group**	CKD	CKD	None	High risk of AKI	Diabetes	CKD
**Mean Age (sd)**	Intervention: 73 (9) Control: 72 (8)	Intervention: 72(10) Control: 72 (10)	65(12)	Intervention: 71(11) Control: 70 (12)	NR	NR
**Female (%)**	26	52	33	31	NR	NR
**AKI definition**	Increase in SCr ≥25% or ≥0.5mg from baseline	Increase in SCr >25% or 0.5mg from baseline	Increase in SCr ≥25% from baseline	Increase in SCr ≥50% or ≥0.3mg from baseline	Increase in SCr ≥25% or ≥0.5mg from baseline	Increase in SCr ≥25% from baseline or ≥0.5mg from baseline
**Comorbidities**	Diabetes (54%), hypertension (47%), congestive heart failure (14%), liver cirrhosis (1%)	Hypotension (97%), diabetes (55%)	Diabetes (50%), unstable angina (62%)	Diabetes (47%), Hypertension (88%), congestive heart failure (23%)	Diabetes (100%)	NR
**Study drug**	ACE/ARB	ACE/ARB	ACE/ARB	ACE/ARB	ACE/ARB	NSAIDs
**Intervention: Timing of hold**	24 hours prior to procedure	Day of procedure	24 hours prior to procedure	Morning of surgery	24hrs before procedure	No details
**Intervention: timing of restart**	Up to 96 hours post procedure	24hrs post procedure	(1) Immediately afterwards; (2) 24 hours after	No details	No details	No details
**Control**	Continued throughout study	Continued throughout study	Continued throughout study	Continued throughout study	Continued throughout study	Continued throughout study
**Risk of Bias**	**Low**	**Some**: randomized by coin toss, no information on allocation concealment. Baseline difference compatable with chance	**Some**; no information on treatment allocation, baseline difference compatable with chance	**Moderate**; controlled for confounding but possibility of residual confounding	**Critical**; no control for confounding	**Not assessed**

* **Available only as CONFERENCE ABSTRACT;** RCT=randomized controlled trial; SCr=Serum creatinine; CKD=chronic kidney disease; AKI =acute kidney infection; ACE= Angiotensin-converting enzyme inhibitors,; ARB= Angiotensin receptor blockers NSAIDs= non-steroidal anti-inflammatory drugs

For peer review only - http://bmjopen.bmj.com/site/about/guidelines.xhtml

Table 2: Summary of outcomes evaluated in single studies

Outcome	Study	Effect Size (95% CI)
Urea (24 hour)	Wolak (2013)[16]	MD=2.17 [-5.22, 9.56]
Diastolic blood pressure (48 hour)	Wolak (2013)[16]	MD=0.30 [-5.01, 5.61]
Systolic blood pressure (48 hour)	Wolak (2013)[16]	MD=-2.10 [-12.98, 8.78]
Hypertensive treatment	Wolak (2013)[16]	RR=0.17 [0.01, 3.69]
Death	Bainey (2015)[18]	RR=3.15 [0.13, 78.17]
Myocardial infarction	Bainey (2015)[18]	No events
Stroke	Bainey (2015)[18]	RR=3.15 [0.13, 78.17]
Congestive heart failure	Bainey (2015)[18]	No events
Rehospitalisation	Bainey (2015)[18]	RR=7.49 [0.38, 146.89]
Interleukin 18 (IL 18) (≥120 ng/mL)	Coca (2013)[19]	0.89 [0.65, 1.23]*
Kidney injury molecule 1 (KIM 1) (≥1.15 ng/mL)	Coca (2013)[19]	1.09 [0.82, 1.44]*
Liver fatty acid binding protein (L-FABP) (≥170 ng/mL)	Coca (2013)[19]	0.97 [0.73, 1.3]*
Neutrophilgelatinase-associated lipocalin (NGAL) (≥120 ng/mL)	Coca (2013)[19]	0.84 [0.60, 1.16]*

* Adjusted for sex, age, white, CKD-EPI eGFR, diabetes, hypertension, congestive heart failure, myocardial infarction, cardiac cauterization in past 48h, electic surgery and type of surgery (CABG, valve, both)

For peer review only

For peer review only - http://bmjopen.bmj.com/site/about/guidelines.xhtml

Table 3: GRADE Evidence Profile: Risks and benefits of temporarily discontinuing medications to prevent acute kidney injury

Quality assessment						№ of patients		Effect		Quality
**Studies**	**Risk of bias**	**Inconsistency**	**Indirectness**	**Imprecision**	**Other considerations**	**Continuation**	**Discontinuation**	**Relative (95% CI)**	**Absolute (95% CI)**	
**Incidence of acute kidney injury**										
3 RCTs	not serious	not serious	not serious	serious [1]	publication bias strongly suspected [2]	27/248 (10.9%)	21/274 (7.7%)	RR 1.48 (0.84 to 2.60)	52 more per 1,000 (from 17 fewer to 174 more)	⊕⊕◯◯ LOW
3 RCTs 3 Cohorts	very serious [3]	not serious	not serious	not serious	publication bias strongly suspected [2]	134/520 (25.8%)	323/1099 (29.4%)	RR 1.14 (0.96 to 1.36)	36 more per 1,000 (from 10 fewer to 93 more)	⊕◯◯◯ VERY LOW

CI: Confidence interval; **RR:** Risk ratio

1. Wide CI and few events
2. Non randomised studies appear would have been unlikely to have been written up for publication if findings had been negative therefore similar studies with negative findings considered likely
3. 3 RCTS, no serious concerns regarding risk of bias. 2 cohort studies, 1 judged moderate risk of bias due to possibility of residual confound, 1 judged critical risk of bias as did not control for confounding

For peer review only - http://bmjopen.bmj.com/site/about/guidelines.xhtml

Appendix 1: Search Strategy Medline

Language: all

Date parameters: all

Search Strategy:

1 ((sick day$ or well day$) adj2 (management or protocol$ or recommendation$ or rule$)).ti,ab. (40)

2 ((drug$ or pill$ or medicin$ or medication$) adj2 holiday$).ti,ab. (396)

3 1 or 2 (436)

4 exp "Angiotensin Receptor Antagonists"/ (17888)

5 ((angiotensin adj3 (receptor$ adj2 (antagonist$ or blocker$))) or arb or arbs).ti,ab. (11806)

6 (candesartan or amias or eprosartan or teveten or irbesartan or aprovel or coaprovel or losartan or cozaar or cozaar-comp or olmesartan or olmetec or sevikar or telmisartan or micardis or valsartan or diovan or co-diovan).ti,ab. (14149)

7 exp Angiotensin-Converting Enzyme Inhibitors/ (39220)

8 ((ace or acei or ((angiotensin adj converting adj2 enzyme*) or ace or kininase)) adj2 (inhibitor* or antagonist*)).ti,ab. (29275)

9 (captopril or ecopace or kaplon or capoten or co-zidocapt or capto-co or capozide or cilazapril or vascace or enalapril or ednyt or innovace or innozide or fosinopril or imidapril or tanatril or lisinopril or zestril or carace or zestoretic or moexipril or perdix or perindopril or coversyl or quinapril or quinil or accupro or accuretic or ramipril or tritace or triapin or trandolapril or gopten or tarka).ti,ab. (22409)

10 (renin adj4 (antagonist$ or blocker$ or inhibitor$)).ti,ab. (3552)

11 aliskiren.ti,ab. (868)

12 exp Diuretics/ (71825)

13 (diuretic$ or thiazide$ or indapamide or chlortalidone or bedroflumethiazide or xipamide or metaolozone or cyclopenthiazide or furosemide or bumetanide or torasemide or amiloride or triamterene or spironalactone or eplerenone or co-amilofruse or co-amilozide or mannitol).ti,ab. (67540)

14 exp Mineralocorticoid Receptor Antagonists/ or aldosterone antagonist$.ti,ab. (8409)

15 exp Anti-Inflammatory Agents, Non-Steroidal/ (161061)

16 (nsaid$ or ibuprofen or naproxen or fenoprofen or ketoprofen or diclofenac or aceclofenac or etodolac or indometacin or mefenamic acid or meloxicam or nabumetone or phenylbutazone or piroxicam or sulindac or tenoxicam or tolenamic acid or etoricoxib or celecoxib or acemetacin or dexibuprofen or dexketoprofen or flurbiprofen or tiaprofenic acid).ti,ab. (50654)

17 aspirin.ti,ab. (38487)

18 Metformin/ (7874)

19 metformin.ti,ab. (10748)

20 exp Sulfonylurea Compounds/ (16449)

21 (sulfonylurea$ or sulphonylurea$ or acetohexamide or carbutamide or chlorpropamide or gliclazide or glipizide or glyburide or tolazamide or tolbutamide or glibenclamide or glimepiride or glibornuride or gliquidone or glisoxepide or glyclopyramide or glimipramide).ti,ab. (20419)

22 or/4-21 (378495)

23 Withholding Treatment/ (9466)

24 (withhold$ or withheld$ or "non use" or withdraw$ or avoid$ or restart$ or continu$ or discontinu$ or stop$ or suspend$ or suspension or ceas$ or cessation).ti,ab. (1375429)

25 or/23-24 (1381746)

26 exp angiotensin ii type 1 receptor blockers/ad, ae, ct, tu, to or angiotensin ii type 2 receptor blockers/ad, ae, tu (6817)

27 exp Angiotensin-Converting Enzyme Inhibitors/ad, ae, ct, tu, to (27160)

28 "Angiotensin Receptor Antagonists"/ad, ae, ct, tu (1189)

29 exp Mineralocorticoid Receptor Antagonists/ad, ae, ct, tu, to (4429)

30 exp Diuretics/ad, ae, ct, tu, th, to or exp Anti-Inflammatory Agents, Non-Steroidal/ad, ae, ct, tu, to or Metformin/ad, ae, ct, tu, to (132402)

31 exp Sulfonylurea Compounds/ad, ae, ct, tu, to (7005)

32 ((ace$ or arb$ or diuretic$ or thiazide$ or nsaid$ or metformin or sulfonylurea$ or sulphonylurea$ or DANS) adj3 (side effect$ or adverse effect$ or adverse event$ or danger$ or injur$ or toxic$ or nephrotoxic$)).ti,ab. (5219)

For peer review only

33 or/26-32 (167054)
34 sepsis/ or exp bacteremia/ or shock, septic/ (81961)
35 (sepsis or septic).ti,ab. (94667)
36 ((toxic or endotoxic) adj shock*).ti,ab. (5911)
37 septic?emi*.ti,ab. (17808)
38 (blood stream adj2 infect*).ti,ab. (794)
39 Diarrhea/ (39641)
40 (diarrhoea* or diarrhea*).ti,ab. (81268)
41 Vomiting/ (19797)
42 (vomit* or emesis).ti,ab. (55475)
43 ((critical or serious or acute or intercurrent or concurrent) adj3 illness$).ti,ab. (20530)
44 Influenza, Human/ or *critical illness/ (47114)
45 influenza.ti,ab. (71591)
46 ((sodium or volume) adj3 depletion).ti,ab. (2605)
47 Dehydration/ (10728)
48 dehydration.ti,ab. (22570)
49 ((urinary or respiratory or skin or viral or bacterial or major or serious) adj4 infection$).ti,ab. (170172)
50 (UTI$ or RTI$).ti,ab. (534252)
51 exp *Surgical Procedures, Operative/ (1498735)
52 (surger$ or surgical or operation or operations or operativ$).ti,ab. (1585555)
53 exp Contrast Media/ (95342)
54 ((contrast$ or radiocontrast$) adj3 (agent$ or material$ or medium or media)).ti,ab. (49004)
55 or/34-54 (3590845)
56 exp Acute Kidney Injury/ (35200)
57 ((acute or early) adj (kidney or renal) adj (failure* or injur* or insufficien* or dysfunction* or impair*)).ti,ab. (31135)
58 (acute adj3 (kidney necrosis or tubul* necrosis)).ti,ab. (2972)
59 AKI.ti,ab. (4409)
60 Kidney Diseases/ci [Chemically Induced] (9718)
61 Renal Insufficiency/ci [Chemically Induced] (1216)
62 or/56-61 (58312)
63 exp *acute kidney injury/pc, ci, co, th (10689)
64 *Kidney Diseases/pc, ci, co (11347)
65 *Renal Insufficiency/pc, ci, co (1991)
66 ((AKI or acute kidney injury or acute renal injury or acute kidney failure or acute renal failure or acute kidney necrosis or acute tubular necrosis or acute kidney tubular necrosis or acute kidney insufficienc$ or acute renal insufficienc$ or acute kidney impair$ or acute renal impair$ or acute kidney dysfunction or acute renal dysfunction) adj3 (adverse event$ or adverse effect$ or mortality or morbidity or death$ or prevent$ or treat or treatment or incidence or caus$ or complication$ or minimiz$)).ti,ab. (6112)
67 or/63-66 (28252)
68 (kidney$ or renal or nephro$).mp. (929261)
69 3 and 68 (20)
70 22 and 25 and 62 (924)
71 22 and 55 and 62 (1468)
72 33 and 67 (2411)
73 69 or 70 or 71 or 72 (3765)
74 letter/ (877999)
75 editorial/ (377501)
76 news/ (168594)
77 exp historical article/ (333817)
78 Anecdotes as topic/ (4624)
79 comment/ (625733)
80 case report/ (1731454)
81 (letter or comment$).ti. (102853)
82 animals/ not humans/ (3943670)

For peer review only

83 exp Animals, Laboratory/ (745711)
84 exp Animal Experimentation/ (6628)
85 exp Models, Animal/ (436993)
86 exp rodentia/ (2737281)
87 (rat or rats or mouse or mice).ti. (1142491)
88 or/74-87 (8050101)
89 73 not 88 (2085)
90 exp child/ or exp infant/ or (child$ or infant$ or neonat$ or newborn$ or baby or babies or p?ediatric).ti. (2239441)
91 exp adult/ or adult$.ti. (5842639)
92 90 not 91 (1539523)
93 89 not 92 (1924)

For peer review only

Section/topic	#	Checklist item	Reported on page #
**TITLE**			
Title	1	Identify the report as a systematic review, meta-analysis, or both.	1
**ABSTRACT**			
Structured summary	2	Provide a structured summary including, as applicable: background; objectives; data sources; study eligibility criteria, participants, and interventions; study appraisal and synthesis methods; results; limitations; conclusions and implications of key findings; systematic review registration number.	2
**INTRODUCTION**			
Rationale	3	Describe the rationale for the review in the context of what is already known.	4
Objectives	4	Provide an explicit statement of questions being addressed with reference to participants, interventions, comparisons, outcomes, and study design (PICOS).	4
**METHODS**			
Protocol and registration	5	Indicate if a review protocol exists, if and where it can be accessed (e.g., Web address), and, if available, provide registration information including registration number.	5
Eligibility criteria	6	Specify study characteristics (e.g., PICOS, length of follow-up) and report characteristics (e.g., years considered, language, publication status) used as criteria for eligibility, giving rationale.	5
Information sources	7	Describe all information sources (e.g., databases with dates of coverage, contact with study authors to identify additional studies) in the search and date last searched.	5
Search	8	Present full electronic search strategy for at least one database, including any limits used, such that it could be repeated.	Web Appendix
Study selection	9	State the process for selecting studies (i.e., screening, eligibility, included in systematic review, and, if applicable, included in the meta-analysis).	6

For peer review only

Section/topic	#	Checklist item	Reported on page #
Data collection process	10	Describe method of data extraction from reports (e.g., piloted forms, independently, in duplicate) and any processes for obtaining and confirming data from investigators.	6
Data items	11	List and define all variables for which data were sought (e.g., PICOS, funding sources) and any assumptions and simplifications made.	6
Risk of bias in individual studies	12	Describe methods used for assessing risk of bias of individual studies (including specification of whether this was done at the study or outcome level), and how this information is to be used in any data synthesis.	6
Summary measures	13	State the principal summary measures (e.g., risk ratio, difference in means).	7
Synthesis of results	14	Describe the methods of handling data and combining results of studies, if done, including measures of consistency (e.g., I^2) for each meta-analysis.	7
Risk of bias across studies	15	Specify any assessment of risk of bias that may affect the cumulative evidence (e.g., publication bias, selective reporting within studies).	7
Additional analyses	16	Describe methods of additional analyses (e.g., sensitivity or subgroup analyses, meta-regression), if done, indicating which were pre-specified.	7
**RESULTS**			
Study selection	17	Give numbers of studies screened, assessed for eligibility, and included in the review, with reasons for exclusions at each stage, ideally with a flow diagram.	7; Figure 1
Study characteristics	18	For each study, present characteristics for which data were extracted (e.g., study size, PICOS, follow-up period) and provide the citations.	8; Table 1
Risk of bias within studies	19	Present data on risk of bias of each study and, if available, any outcome level assessment (see item 12).	8; Table 1

For peer review only

Section/topic	#	Checklist item	Reported on page #
Results of individual studies	20	For all outcomes considered (benefits or harms), present, for each study: (a) simple summary data for each intervention group (b) effect estimates and confidence intervals, ideally with a forest plot.	Figure 2
Synthesis of results	21	Present results of each meta-analysis done, including confidence intervals and measures of consistency.	Figure 2; Table 2
Risk of bias across studies	22	Present results of any assessment of risk of bias across studies (see Item 15).	Table 3
Additional analysis	23	Give results of additional analyses, if done (e.g., sensitivity or subgroup analyses, meta-regression [see Item 16]).	Figure 2
**DISCUSSION**			
Summary of evidence	24	Summarize the main findings including the strength of evidence for each main outcome; consider their relevance to key groups (e.g., healthcare providers, users, and policy makers).	Table 3; 11
Limitations	25	Discuss limitations at study and outcome level (e.g., risk of bias), and at review-level (e.g., incomplete retrieval of identified research, reporting bias).	12
Conclusions	26	Provide a general interpretation of the results in the context of other evidence, and implications for future research.	15
**FUNDING**			
Funding	27	Describe sources of funding for the systematic review and other support (e.g., supply of data); role of funders for the systematic review.	16

For peer review only

What are the risks and benefits of temporarily discontinuing medications to prevent acute kidney injury? A Systematic Review and meta-analysis

Journal:	*BMJ Open*
Manuscript ID	bmjopen-2016-012674.R1
Article Type:	Research
Date Submitted by the Author:	21-Sep-2016
Complete List of Authors:	Whiting, Penny; The National Institute for Health Research Collaboration for Leadership in Applied Health Research and Care West (NIHR CLAHRC West) at University Hospitals Bristol NHS Foundation Trust; University of Bristol, School of Social and Community Medicine<br>Morden, Andrew; The National Institute for Health Research Collaboration for Leadership in Applied Health Research and Care West (NIHR CLAHRC West) at University Hospitals Bristol NHS Foundation Trust; University of Bristol, School of Social and Community Medicine<br>Tomlinson, Laurie; UK Renal Registry; London School of Hygiene and Tropical Medicine<br>Caskey, Fergus; University of Bristol; UK Renal Registry<br>Blakeman, Thomas; University of Manchester, School of Community Based Medicine; National Institute for Health Research Collaboration for Leadership in Applied Health Research and Care (NIHR CLAHRC) Greater Manchester, Centre for Primary Care, Institute of Population Health<br>Tomson, Charles; Freeman Hospital, Department of Renal Medicine<br>Stone, Tracey; The National Institute for Health Research Collaboration for Leadership in Applied Health Research and Care West (NIHR CLAHRC West) at University Hospitals Bristol NHS Foundation Trust; University of Bristol, School of Social and Community Medicine<br>Richards, Alison; The National Institute for Health Research Collaboration for Leadership in Applied Health Research and Care West (NIHR CLAHRC West) at University Hospitals Bristol NHS Foundation Trust; University of Bristol, School of Social and Community Medicine<br>Savovic, Jelena; University of Bristol, NIHR CLAHRC West; University of Bristol, School of Social and Community Medicine<br>Horwood, Jeremy; The National Institute for Health Research Collaboration for Leadership in Applied Health Research and Care West (NIHR CLAHRC West) at University Hospitals Bristol NHS Foundation Trust; University of Bristol, UK, School of Social and Community Medicine
**Primary Subject Heading**:	Renal medicine
Secondary Subject Heading:	Evidence based practice
Keywords:	Acute kidney injury, Medication discontinuation, Sick day rules, Angiotensin-converting enzyme inhibitors, Angiotensin receptor blockers, NSAIDs

SCHOLARONE™
Manuscripts

For peer review only

What are the risks and benefits of temporarily discontinuing medications to prevent acute kidney injury? A Systematic Review and meta-analysis

Penny Whiting[1,2,], Andrew Morden[1,2*], Laurie A Tomlinson[3,4], Fergus Caskey[2,3], Thomas Blakeman[5,6], Charles Tomson[7], Tracey Stone[1,2], Alison Richards[1,2], Jelena Savović[1,2], Jeremy Horwood[1,2]

* Corresponding Author: penny.whiting@bristol.ac.uk
University Hospitals Bristol NHS Foundation Trust, 9th Floor, Whitefriars, Lewins Mead, Bristol BS1 2NT, Tel. +44 117 34 212 73

[1]The National Institute for Health Research Collaboration for Leadership in Applied Health Research and Care West (NIHR CLAHRC West) at University Hospitals Bristol NHS Foundation Trust, Bristol, UK

[2]School of Social and Community Medicine, University of Bristol, Bristol, UK

[3]UK Renal Registry, Bristol, UK

[4]Department of Non-communicable Disease Epidemiology, London School of Hygiene and Tropical Medicine, London, UK

[5]Centre for Primary Care, Institute of Population Health, The University of Manchester, Manchester, UK

[6]National Institute for Health Research Collaboration for Leadership in Applied Health Research and Care (NIHR CLAHRC) Greater Manchester, Centre for Primary Care, Institute of Population Health, University of Manchester, Manchester, UK

[7]Department of Renal Medicine, Freeman Hospital, Newcastle Upon Tyne Hospitals Foundation Trust, Tyne and Wear, UK

For peer review only

Keywords:

Acute kidney injury, Medication discontinuation, Sick day rules, Angiotensin-converting enzyme inhibitors, Angiotensin receptor blockers, NSAIDs

Word count: Article: 3483; Abstract: 295; Figures: 4; Table: 3

For peer review only - http://bmjopen.bmj.com/site/about/guidelines.xhtml

Abstract

Objectives: To summarise evidence on temporary discontinuation of medications to prevent acute kidney injury (AKI).

Design: Systematic review and meta-analysis of randomized and non-randomized studies.

Participants: Adults taking diuretics, angiotensin-converting-enzyme **(ACE)** inhibitors, angiotensin receptor blockers (ARB), direct renin inhibitors, non-steroidal anti-inflammatories, metformin or sulfonylureas, experiencing inter-current illnesses, radiological or surgical procedures.

Interventions: Temporary discontinuation of any of the medications of interest.

Primary and secondary outcome measures: Risk of AKI. Secondary outcome measures were estimated glomerular filtration rate (eGFR) and creatinine post AKI, urea, systolic and diastolic blood pressure, death, clinical outcomes and biomarkers.

Results: Six studies were included (1,663 participants), three randomised controlled trials (RCTs) and three prospective cohort studies. Mean age ranged from 65 to 73 years, the proportion of women ranged from 31 to 52%. All studies were in hospital settings; five evaluated discontinuation of medication prior to coronary angiography and one prior to cardiac surgery. Five studies evaluated discontinuation of ACE inhibitors and ARBs, one small cohort study looked at discontinuation of NSAIDS. No studies evaluated discontinuation of medication in the community following an acute inter-current illness. There was an increased risk of AKI of around 15% in those in whom medication was continued compared to those in whom it was discontinued (relative risk (RR) 1.17, 95% CI 0.99, 1.38; 5 studies). When only results from RCTs were pooled, the increase in risk was almost 50% (RR 1.48, 95% CI 0.84, 2.60; 3 RCTs) but the confidence interval was wider. There was no difference between groups for any secondary outcomes.

Conclusions: There is low quality evidence that withdrawal of ACE inhibitors/ARBs prior to coronary angiography and cardiac surgery may reduce the incidence of AKI. There is no evidence of the impact of drug cessation interventions on AKI incidence during inter-current illness in primary or secondary care.

For peer review only - http://bmjopen.bmj.com/site/about/guidelines.xhtml

Systematic review registration: PROSPERO CRD42015023210

Article summary

Strengths and limitations of this study:

- We have conducted a thorough systematic review of the evidence from studies that have examined interventions involving temporary discontinuation of medications to prevent or minimise the severity, or consequences, of AKI.
- This is a topic of major importance due to interventions currently being implemented to reduce the risk of AKI throughout the UK and internationally
- Broad eligibility criteria included both randomized and non-randomized studies; Primary and secondary care; inter-current illness or a radiological/surgical procedure; planned and unplanned settings.
- The strength of the conclusion is limited by the quality and number of studies, and absence of evidence for important settings and classes of medications.

For peer review only

Background

Acute kidney injury (AKI) is a sudden decline in renal function, affecting up to 20% of people admitted to hospital, and is strongly associated with increased mortality and longer duration of hospital stay.[1] Historically recognition and treatment of AKI has been poor.[2] Recent comprehensive initiatives in the UK have focussed on improving awareness and treatment of people with or at risk of AKI.[3] It is thought that a substantial proportion of AKI is triggered or exacerbated by prescribed medications, particularly during times of physiological stress such as inter-current illness, surgery or radiocontrast imaging. [4] These medications include angiotensin-converting-enzyme inhibitors (ACEI), Angiotensin Receptor Blockers (ARB), diuretics, non-steroidal anti-inflammatory drugs (NSAIDs). Under the same circumstances reduced excretion of metformin is associated with an increased risk of lactic acidosis while sulfonylureas can lead to a greater incidence of hypoglycaemia. Therefore, many clinicians, expert consensus statements and guidelines recommend that some or all of these medications are stopped prior to elective or emergency procedures, or when patients become unwell with symptoms of severe infection. [5 6] Initiatives advising patients prescribed these medications to temporarily stop taking them when they become unwell (so called 'sick-day rules') have been implemented throughout Scotland and in local initiatives across the United Kingdom (UK). [7] However, the evidence base to support these recommendations is unclear, and the overall benefit remains controversial. [8]

We conducted a systematic review and meta-analysis of the randomised and non-randomized studies that have examined temporary discontinuation of all or any of these medications in patients in primary or secondary care at risk of AKI or with newly diagnosed AKI as a result of an inter-current illness or a radiological/surgical procedure (planned or unplanned).

For peer review only - http://bmjopen.bmj.com/site/about/guidelines.xhtml

Methods

Systematic review methods followed guidance from the Centre for Reviews and Dissemination (CRD) [9] and the Cochrane Collaboration; [10] this review is reported according to the PRISMA guidelines. [11] The review followed a predefined published protocol. [12]

Study Eligibility criteria

Studies, both randomized and non-randomized, that evaluated adults (age ≥18 years) who were taking a specified medication and experiencing an inter-current illness or undergoing a radiological/surgical procedure (planned or unplanned) in whom the medication was temporarily discontinued for any reason were eligible for inclusion. Medications of interest were diuretics, ACEIs, ARBs, direct renin inhibitors, NSAIDs, metformin or sulfonylureas. Studies had to report a measure of kidney function (e.g. incidence of AKI, estimated glomerular filtration rate (eGFR), or serum creatinine) and include a comparator group consisting of placebo, no treatment or usual care.

Identification and selection of studies

The following databases were searched from inception to January 2016: Embase, Medline, PsycINFO, BIOSIS Citation Index (Web of Science), CINAHL (Cumulative Index to Nursing and Allied Health Literature), Science Citation Index (SCI) (Web of Science), and the Cochrane Central Register of Controlled Trials (CENTRAL). Supplementary searches were undertaken to identify grey literature, completed and ongoing trials, in the following resources: NIH ClinicalTrials.gov (http://www.clinicaltrials.gov), metaRegister of Controlled Trials (http://www.controlled-trials.com), WHO International Clinical Trials Registry Platform (ICTRP) (http://www.who.int/ictrp/en), relevant guidelines (e.g. NICE in the UK) regarding management of AKI. Reference lists of included studies were screened. Details of the Medline search strategy is available as a supplementary appendix.

For peer review only - http://bmjopen.bmj.com/site/about/guidelines.xhtml

For peer review only

Search results and full-text articles were independently assessed for inclusion by two reviewers; disagreements were resolved through consensus or referral to a third reviewer where necessary.

Data extraction and assessment of risk of bias

We extracted data on baseline characteristics (number of participants, participant characteristics, study settings, study design, country, inclusion and exclusion criteria), intervention/exposure related to stopping medication, and outcomes. The primary outcome was iincidence of AKI secondary outcomes included urinary biomarkers, clinical outcomes, creatinine, eGFR, urea and blood pressure. For dichotomous data (for example incidence of AKI) we extracted the number of events and participants in each treatment group and calculated the relative risk (RR) and 95% confidence interval (CI). For continuous data, we extracted the mean and standard deviation in each treatment group and calculated mean differences (MD) and 95% CIs.

RCTs were assessed for methodological quality using a draft version of the new Cochrane risk of bias tool[13] that includes items covering allocation bias (random sequence generation, allocation concealment and baseline imbalance), departures from interventions (participant and study personnel blinding, deviations from intended interventions and analysis in groups to which they were randomized), attrition bias (incomplete outcome data and robustness of results to missing data) detection bias (blinding of outcome assessors and likelihood of blinding to have influenced results), and reporting bias (selective reporting of outcome domain being assessed). The ROBINS-I tool was used to assess the risk of bias in non-randomized studies.[14] It includes domains covering bias due to confounding, bias in the selection of participants into the study, bias due to departures from intended interventions, bias due to missing data, bias in taking measurements and bias in the selection of the reported result.

For peer review only - http://bmjopen.bmj.com/site/about/guidelines.xhtml

Data was extracted by one reviewer using a standard data extraction form designed for this review, and checked by a second reviewer. Risk of bias assessment was performed independently by two reviewers. Any disagreements were resolved by consensus or referral to a third reviewer.

Data synthesis

We grouped studies by design (randomized versus non-randomized), population (coronary angiography versus surgery) and outcome. If there were two or more studies assessing the same outcome, data were plotted on a forest plot. If data were considered statistically and clinically sufficiently homogeneous then summary estimates were produced using random effects meta-analysis. When the same outcomes were assessed in both randomized and non-randomized studies that were considered similar in terms of population and intervention, we first stratified the analysis based on study design. If summary estimates from stratified analyses were considered sufficiently similar we then produced an overall summary estimate combining data from randomized and non-randomized studies. For dichotomous outcomes we estimated summary RRs and 95% CIs, for continuous data we estimated summary MDs and 95% CIs. Heterogeneity was investigated using forest plots and the I^2 statistic. Where data were considered too heterogeneous to pool, a narrative synthesis was provided. We used GRADE to rate the overall quality of the evidence for risk of bias, publication bias, imprecision, inconsistency, indirectness, and magnitude of effect.[15]

Results

Search results

The searches identified 4316 hits (records) of which 42 were considered potentially relevant and obtained for full text review (Figure 1). A total of 6 studies (1663 participants) were included in the review: three RCTs (522 participants)[16-18] and three prospective cohort studies (1141 participants).[19-

For peer review only - http://bmjopen.bmj.com/site/about/guidelines.xhtml

[21] One study was available only as a conference abstract and so limited details were available for this study.[21]

All studies were conducted in hospital settings: five evaluated discontinuation of medication prior to coronary angiography and one prior to cardiac surgery.[19] All but one study[16] restricted inclusion to patients deemed at higher risk of AKI such as those with chronic kidney disease (3 studies),[17 18 20] diabetes (1 study),[21] or a set of criteria that defined patients at high risk (1 study).[19] The most commonly reported co-morbidities included diabetes, hypertension and congestive heart failure. No studies of discontinuation of medication in the community following an acute inter-current illness were found. Studies were conducted in North America, Turkey and Israel. Mean age, where reported, ranged from 65 to 73 years and the proportion of women in the studies ranged from 31 to 52%. Five studies evaluated discontinuation of ACE inhibitors and ARBs, one small cohort study looked at discontinuation of NSAIDs.[20] The time point at which the medication was stopped varied between studies. Three studies reported that medication was stopped 24 hours prior to the procedure,[16 18 21] two (including the one study of surgery) that it was stopped on the morning of the procedure,[17 19] and one (the study of NSAIDs)[20] did not provide details on when medication was stopped. The time point at which medication was started again also varied. One study stated that medication was started up to 96 hours post-procedure[18], one RCT[16] included two intervention arms that compared restarting immediately post-procedure with restarting 24 hours after the procedure and four studies did not report on this. No studies were found that assessed discontinuation of diuretics, metformin or sulfonylureas. Table 1 provides an overview of included studies.

Risk of bias

The risk of bias assessment was performed for the primary outcome of incidence of AKI. Two RCTs were judged to have 'some concerns' regarding risk of bias[16 17] and one was rated 'low risk of bias'.[18] The two judged at 'some concern' both had issues with the randomization process; all other bias

For peer review only - http://bmjopen.bmj.com/site/about/guidelines.xhtml

domains were rated 'low risk'. One provided no information on the methods used to allocate participants to the two treatment groups, the other randomized patients by means of a coin toss but did not provide any information on whether allocation was concealed. Both studies provided a reasonable overview of baseline characteristics, including similarities in timings of baseline kidney function, which suggested that any differences between groups were compatable with chance. The risk of bias assessment highlighted that none of the studies provided information on blinding of participants, study personnel or outcome assessors. However, there do not appear to have been any departures from the intended interventions thus knowledge of the assigned intervention appears unlikely to have influenced the study result. The outcome measure was considered relatively objective and therefore also unlikely to have been influenced by knowledge of treatment assignment.

It was not possible to conduct a risk of bias assessment for one of the non-randomized studies[20] as this study did not provide any numerical data and the risk of bias assessment is performed at the result level. One of the non-randomized studies was judged at moderate risk of bias,[19] the other at critical risk of bias.[21] The study judged at critical risk of bias only presented crude outcome data with no adjustment for potential confounding factors. It was judged at low risk of bias for all other domains with the exception of measurement of interventions which was judged at moderate risk of bias as it was not clear exactly how exposure to ACE inhibitors and ARBs was measured. The study judged at moderate risk of bias was judged to have appropriately controlled for confounding factors but the guidance for the ROBINS-I tool states that this domain can only be rated as low risk of bias if the study is considered comparable to a well-perfomed randomized trial.

Incidence of AKI

One study did not provide any numerical data on the effect of discontinuation of medication on patient outcomes.[20] This cohort study, which assessed discontinuation of NSAIDs, only found three

For peer review only - http://bmjopen.bmj.com/site/about/guidelines.xhtml

patients out of 44 NSAID users who were advised to discontinue their medication prior to coronary angiography. It reported that discontinuation of NSAID was not associated with a lower rate of AKI, but this was limited by the small number of patients in whom medication was discontinued.

All other studies assessed the incidence of AKI (or contrast induced nephropathy) in those in whom medication was stopped prior to the procedure compared to those in whom medication was continued (Figure 2). Three studies defined AKI as an increase in creatinine of 25% or 0.5 mg/dL above baseline,[17 18 21] one as an increase in creatinine of 25% above baseline,[16] and one used a slightly different definition of an increase in creatinine of 50% or 0.3mg/dL above baseline.[19] All but one suggested an increased risk of AKI in those in whom medication was continued, but confidence intervals were generally wide. There was an increased risk of AKI of around 15% in those in whom medication was continued compared to those in whom it was discontinued (RR 1.17, 95% CI 0.99, 1.38). Omitting the study judged at critical risk of bias had very little effect on the summary estimate (RR 1.16, 95% CI 0.98, 1.37). When only results from RCTs were pooled, the increase in risk was almost 50% (RR 1.48, 95% CI 0.84, 2.60; 3 RCTs) but the confidence interval was much wider. There was no evidence of heterogeneity for any of these analyses (I^2=0%). Based on GRADE the quality of the evidence was judged as low for the analysis restricted to RCTs and very low when non-randomized studies were included (Table 3). The evidence was downgraded due to imprecision and the likelihood of publication bias for the analysis that included RCTs and for study quality and publication bias for the analysis that included non-randomized trials.

Secondary outcomes

Two studies[16 17] assessed GFR and creatinine at 24 hours post-intervention (Figures 3 and 4). Both suggested no difference in these measures between intervention groups, although confidence intervals were wide. Other outcomes reported in single studies included urinary biomarkers (structural AKI), clinical endpoints (mortality, myocardial infarction, stroke, congestive heart failure,

For peer review only - http://bmjopen.bmj.com/site/about/guidelines.xhtml

rehospitalisation, hypertensive treatment) and blood pressure. Generally there was no difference between groups in which medication was stopped and groups in which it was continued for any of these outcomes. Table 2 provides an overview of key outcomes; other outcomes reported in included studies were different ways of measuring these outcomes (e.g. continuous rather than dichotomous data, or change from baseline rather than absolute value).

Discussion

The results of our meta-analysis demonstrate an approximately 15% increased risk of AKI in those in whom medication was continued compared to those in whom it was discontinued (RR 1.17, 95% CI 0.99, 1.38). When only results from RCTs were pooled, the increase in risk was almost 50% (RR 1.48, 95% CI 0.84, 2.60) but the confidence interval was much wider. Based on the GRADE approach, the quality of the evidence was low when restricted to RCTs and very low when non-randomized studies were included. There was no difference between groups in which medication was stopped and groups in which it was continued for any secondary outcomes but these were mainly assessed in single studies.

This is the first systematic review into a topic of major importance, as interventions of this type are currently being implemented throughout the UK and internationally, with the aim of reducing the incidence and/or severity of AKI. We have used broad inclusion criteria in many databases to capture randomised and non-randomized studies, in primary and secondary care, and for a range of AKI precipitants including inter-current illness and planned or emergency radiological and surgical procedure. However, we have found that the published evidence was sparse and has important limitations. It is focussed in hospital settings, mainly in patients undergoing coronary angiography, restricted to patients who were considered high risk for AKI and predominantly evaluates discontinuation of ACE inhibitors and ARBs. The primary definition of AKI in all studies was based on

For peer review only - http://bmjopen.bmj.com/site/about/guidelines.xhtml

short-term changes in serum creatinine although definitions varied across studies. The definitions of AKI used in four of the studies may have overestimated the incidence of AKI compared to the currently accepted definition of AKI, which was used in only one study.[19]

Since ACEIs/ARBs reduce glomerular filtration rate but preserve tubular blood flow, a more marked short-term reduction in eGFR may be associated with lower rates of established AKI due to ongoing tubular injury.[22] Indeed, the only study[19] that examined alternate biomarker-based definitions of AKI found no effect related to drug cessation. In addition, the longer term impact of AKI in terms of the development of CKD or reductions in baseline GFR was not reported. The reduction in glomerular filtration rate caused by ACEI and ARB treatment is reversible on stopping the drug.[23] This temporary rise in GFR among patients who discontinued the drugs might have masked AKI in the studies included here, given that AKI was defined as a change in serum creatinine from a baseline measurement that was taken prior to drug withdrawal. Recognising the potential for physiological rather than pathological changes in kidney function,[22] future studies will benefit from examining later clinical outcomes including incomplete recovery from AKI (i.e. failure of serum creatinine concentration to return to baseline), chronic kidney disease, and all-cause mortality.

To further quantify the limitations of the studies, we conducted a formal risk of bias assessment using the most recently developed tools. This is the first review to have used both the ROBINS-I tool[14] and the new Cochrane tool for randomised trials.[13] The majority of studies were small and there were some concerns regarding risk of bias in some studies, especially one of the non-randomized studies which was judged at critical risk of bias. Publication bias was not formally assessed in this review because the number of studies was too small for such an assessment to be meaningful. However, our search strategy included a variety of routes to identify unpublished studies and resulted in the inclusion of one conference abstract. Despite this we consider the likelihood of publication bias in this area to be high.

For peer review only - http://bmjopen.bmj.com/site/about/guidelines.xhtml

Importantly, there are no studies which evaluate the benefits of stopping medication in the community following acute infection, and no studies that assessed discontinuation of diuretics that could exacerbate AKI, or metformin and/or sulfonylureas, which may accumulate during an episode of AKI. Only one study assessed discontinuation of NSAIDs and only a very small number of patients discontinued these drugs in this study and so it was not possible to draw conclusions regarding the effects of discontinuing NSAIDs.

At present a number of national organisations provide guidance about medication cessation, as well as many regional schemes and guidelines. The UK NICE guidance published in 2013 recommends consideration of temporarily stopping ACE inhibitors and ARBs in adults having iodinated contrast agents if they have chronic kidney disease with an eGFR less than 40 ml/min/1.73 m^2, and in adults, children and young people with diarrhoea, vomiting or sepsis.[6] In 2015, NHS Scotland and the Scottish Patient Safety Programme initiated a more wide ranging medication cessation intervention. Predominantly via community pharmacists, patients are issued with Sick-Day Rules cards, advising them to stop taking ACEIs/ARBs, NSAIDS, diuretics and metformin when they become unwell with vomiting or diarrhoea, and/or fevers sweats and shaking.[24] Under similar circumstances, guidance from the Canadian Diabetes Association Clinical Practice Guidelines for Chronic Kidney Disease in Diabetes recommends physicians and patients to withhold ACEIs, ARBs, NSAIDs, diuretics, metformin, direct renin inhibitors and Sulfonylureas.[25] This guidance is based on the commonly-held belief that there is an association between the use of ACE Inhibitors/ARBs, diuretics and NSAIDs and the development of AKI, particularly during illness or other physiological insult. The potentially strongest source of evidence, the incidence of AKI in randomised controlled trials of ACEIs and ARBs compared to placebo is poorly described due to variable definitions or absent reporting of kidney related adverse events.[26] A number of observational studies have demonstrated a higher risk of AKI among patients among ACEI/ARB users also taking diuretics and/or NSAIDs compared to those

For peer review only - http://bmjopen.bmj.com/site/about/guidelines.xhtml

taking ACEIs/ARBs alone,[27-29] or with ACEI/ARB users compared to non-users during acute illness or after surgery.[30 31] As with all observational evidence, these studies carry an inherent risk of associations being due to bias and confounding, particularly confounding by indication, in which patients at higher risk of AKI are more likely to be treated with the drugs of interest making a direct causal effect uncertain.

Only one of the studies[17] considered in this review was available at the time of development of the NICE guidance for AKI. [6] The guideline development group discuss explicitly the difficulty of issuing guidance regarding medication cessation (for ACEIs/ARBs only) despite limited evidence.[6] They felt that the available evidence for discontinuation was weak but that the “continuing use of ACEIs/ARBs [during acute illness or exposure to iodinated contrast agents] is clearly associated with AKI. In contrast, the temporary suspension of ACEIs/ARBs for a short period seems unlikely to greatly increase the risk of cardiovascular events.” Subsequent evidence regarding the safety of community medication cessation interventions has come from an ongoing evaluation of hospital admissions following introduction of the NHS Scotland scheme, which has shown a stabilisation or fall in hospital admissions with AKI. [24] However, a concurrent fall in heart failure admissions (which might have been expected to increase as a consequence of discontinuation of ACEIs or ARBs amongst patients previously stabilised on these drugs for treatment of heart failure), suggest a secular trend in hospital admissions unrelated to the introduction of the intervention, and interpretation is also limited by the absence of a control population. There remains ongoing disagreement about how the general evidence base should be interpreted to consider the balance of risks and benefits of drug-cessation interventions, particularly during acute illness.[8]

This systematic review includes five additional studies published since the NICE guidance on AKI. Our results show low quality evidence that withdrawal of ACE Inhibitors/ARBs and NSAIDs prior to coronary angiography and cardiac surgery may reduce the incidence of AKI. However, the quality,

For peer review only - http://bmjopen.bmj.com/site/about/guidelines.xhtml

power and limited scope of these studies reduce the emphasis that can be placed on this finding and have not substantially clarified the evidence base. There is no published evidence of the impact of drug cessation interventions on AKI incidence during inter-current illness in primary or secondary care, of other included medications (NSAIDs, diuretics, sulfonylureas, metformin) or of combinations of medications. We also found no evidence of ongoing studies of interventions on any of these topics.

The current widespread promotion of 'sick-day guidance' incurs financial and opportunity costs. While the public health impact of sick-day guidance can be evaluated through the novel data flows recently established by NHS England and the UK Renal Registry,[3] more formal controlled evaluation in the form of stepped wedge or cluster randomised trials could be applied to ensure we achieve maximal overall public health benefit.

For peer review only

Funding

This research is supported by the National Institute for Health Research (NIHR) Collaboration for Leadership in Applied Health Research and Care (CLAHRC) West at University Hospitals Bristol NHS Foundation Trust. Tom Blakeman was partly funded by the NIHR CLAHRC Greater Manchester. The funders had no role in the design of the study, data collection and analysis, decision to publish, or preparation of the manuscript. However, the project outlined in this article may be considered to be affiliated to the work of the NIHR CLAHRC Greater Manchester and NIHR CLAHR West. The views expressed in this article are those of the authors and not necessarily those of the NHS, NIHR or the Department of Health. Laurie Tomlinson is funded by a Wellcome Trust intermediate clinical fellowship (101143/Z/13/Z).

Competing interests

The authors declare that they have no competing interests.

Authors' contributions

CT, TB and FC conceived the idea for the review. PW, AM, JH and LT drafted the article with the support of FC. AR developed the search strategy. FC, LT, TB and CT served as a content experts in the field of AKI. JH served as the overall supervisor and provided input on study methodology. JS provided methodological support. AM, PW and TS undertook screening and data extraction. PW and FC performed the risk of bias assessment. PW and JS performed the GRADE assessment. All authors contributed to the interpretation of results, commented on draft manuscripts and have given their approval for publication.

For peer review only

Acknowledgements

We would like to thank Dr Tim Jones, NIHR CLAHRC West, for help with assessing the risk of bias in the included studies.

Data sharing statement

No additional data are available.

For peer review only

For peer review only - http://bmjopen.bmj.com/site/about/guidelines.xhtml

References

1. Chertow GM, Burdick E, Honour M, et al. Acute kidney injury, mortality, length of stay, and costs in hospitalized patients. J Am Soc Nephrol 2005;**16**(11):3365-70.
2. National Confidential Enquiry into Patient Outcome and Death. Acute Kidney Injury: Adding Insult to Injury. 2009. http://www.ncepod.org.uk/2009aki.html.
3. Think Kidneys. Acute Kidney Injury: The NHS campaign to improve the care of people at risk of, or with, acute kidney injury. 2016; (11/4/2016). https://www.thinkkidneys.nhs.uk/aki/.
4. Pannu N, Nadim MK. An overview of drug-induced acute kidney injury. Crit Care Med 2008;**36**(4 Suppl):S216-23.
5. Feehally J, Gilmore I, Barasi S, et al. RCPE UK consensus conference statement: Management of acute kidney injury: the role of fluids, e-alerts and biomarkers. The journal of the Royal College of Physicians of Edinburgh 2013;**43**(1):37-8.
6. National Institute for Health and Clinical Excellence. Acute kidney injury: prevention, detection and management. NICE guideline (CG169), 2013.
7. Scottish Patient Safety Programme. Medicine Sick Day Rules Card. 2015. http://www.scottishpatientsafetyprogramme.scot.nhs.uk/programmes/primary-care/medicine-sick-day-rules-card.
8. Griffith K AC, Blakeman T, Fluck R, Lewington A, Selby N, Tomlinson L, Tomson C. “Sick day rules” in patients at risk of Acute Kidney Injury: an Interim Position Statement from the Think Kidneys Board. 2015. https://www.thinkkidneys.nhs.uk/wp-content/uploads/2015/07/Think-Kidneys-Sick-Day-Rules-160715.pdf.
9. Centre for Reviews and Dissemination. Systematic Reviews: CRD’s guidance for undertaking reviews in health care [Internet]. 2009 http://www.york.ac.uk/inst/crd/SysRev/!SSL!/WebHelp/SysRev3.htm.
10. Higgins JPT, Green S. Cochrane handbook for systematic reviews of interventions [Internet]. 2011 http://www.cochrane-handbook.org/.
11. Moher D, Liberati A, Tetzlaff J, et al. Preferred reporting items for systematic reviews and meta-analyses: the PRISMA statement. Ann Intern Med 2009;**151**(4):264-9, w64.
12. Morden A, Horwood J, Whiting P, et al. The risks and benefits of patients temporarily discontinuing medications in the event of an intercurrent illness: a systematic review protocol. Systematic reviews 2015;**4**:139.
13. Savovic J HJ, Sterne J, Boutron I, Hrobjartsson A. Introducing a revised risk of bias tool for randomized trials. Cochrane Colloquium 2015. http://www.cochranelibrary.com/dotAsset/5bc8d6fd-0604-4bed-beb2-8bc7a74aa4b9.pdf.
14. The ROBINS-I tool (Risk Of Bias In Non-randomized Studies - of Interventions). 2016; (16/5/2016). www.riskofbias.info.
15. Guyatt GH, Oxman AD, Vist GE, et al. GRADE: an emerging consensus on rating quality of evidence and strength of recommendations. BMJ 2008;**336**(7650):924-6.
16. Wolak T, Aliev E, Rogachev B, et al. Renal safety and angiotensin II blockade medications in patients undergoing non-emergent coronary angiography: a randomized controlled study. Isr Med Assoc J 2013;**15**(11):682-7.
17. Rosenstock JL, Bruno R, Kim JK, et al. The effect of withdrawal of ACE inhibitors or angiotensin receptor blockers prior to coronary angiography on the incidence of contrast-induced nephropathy. Int Urol Nephrol 2008;**40**(3):749-55.
18. Bainey KR, Rahim S, Etherington K, et al. Effects of withdrawing vs continuing renin-angiotensin blockers on incidence of acute kidney injury in patients with renal insufficiency undergoing cardiac catheterization: Results from the Angiotensin Converting Enzyme Inhibitor/Angiotensin Receptor Blocker and Contrast Induced Nephropathy in Patients Receiving Cardiac Catheterization (CAPTAIN) trial. Am Heart J 2015;**170**(1):110-6.

For peer review only - http://bmjopen.bmj.com/site/about/guidelines.xhtml

19. Coca SG, Garg AX, Swaminathan M, et al. Preoperative angiotensin-converting enzyme inhibitors and angiotensin receptor blocker use and acute kidney injury in patients undergoing cardiac surgery. Nephrol Dial Transplant 2013;**28**(11):2787-99.
20. Weisbord SD, Mor MK, Resnick AL, et al. Prevention, incidence, and outcomes of contrast-induced acute kidney injury. Arch Intern Med 2008;**168**(12):1325-32.
21. Goksuluk H, Kerimli N, Atmaca Y, et al. Effects of renin-angiotensin-aldosterone system blockers on contrast-induced nephropathy and its association with NGAL levels in diabetic patients undergoing coronary angiography. Eur Heart J 2015;**36**:642.
22. Perazella MA, Coca SG. Three feasible strategies to minimize kidney injury in 'incipient AKI'. Nat Rev Nephrol 2013;**9**(8):484-90.
23. Bakris GL, Weir MR. Angiotensin-converting enzyme inhibitor-associated elevations in serum creatinine: is this a cause for concern? Arch Intern Med 2000;**160**(5):685-93.
24. Morrison C, Wilson M. Medicine sick day rules cards: A safe and effective tool to improve medicines safety in NHS Highland. International Journal of Pharmacy Practice 2015;**23**:92-93.
25. Canadian Diabetes Association. Sick Day Medication List. 2015. http://guidelines.diabetes.ca/browse/appendices/appendix7_2015.
26. Palmer SC, Mavridis D, Navarese E, et al. Comparative efficacy and safety of blood pressure-lowering agents in adults with diabetes and kidney disease: a network meta-analysis. Lancet 2015;**385**(9982):2047-56.
27. Dreischulte T, Morales DR, Bell S, et al. Combined use of nonsteroidal anti-inflammatory drugs with diuretics and/or renin-angiotensin system inhibitors in the community increases the risk of acute kidney injury. Kidney Int 2015;**88**(2):396-403.
28. Lapi F, Azoulay L, Yin H, et al. Concurrent use of diuretics, angiotensin converting enzyme inhibitors, and angiotensin receptor blockers with non-steroidal anti-inflammatory drugs and risk of acute kidney injury: nested case-control study. BMJ 2013;**346**:e8525.
29. Loboz KK, Shenfield GM. Drug combinations and impaired renal function -- the 'triple whammy'. Br J Clin Pharmacol 2005;**59**(2):239-43.
30. Arora P, Rajagopalam S, Ranjan R, et al. Preoperative use of angiotensin-converting enzyme inhibitors/angiotensin receptor blockers is associated with increased risk for acute kidney injury after cardiovascular surgery. Clin J Am Soc Nephrol 2008;**3**(5):1266-73.
31. Plataki M, Kashani K, Cabello-Garza J, et al. Predictors of acute kidney injury in septic shock patients: an observational cohort study. Clin J Am Soc Nephrol 2011;**6**(7):1744-51.

For peer review only

For peer review only - http://bmjopen.bmj.com/site/about/guidelines.xhtml

Figure 1 Flow of studies through the review process

Figure 2: Forest plot showing risk of AKI in those who stopped medication prior to procedure compared to those who continued medication

Figure 3: Forest plot showing mean difference in GFR at 24 hours in those who stopped medication prior to procedure compared to those who continued medication

Figure 4: Forest plot showing mean difference in creatinine at 24 hours in those who stopped medication prior to procedure compared to those who continued medication

For peer review only

For peer review only - http://bmjopen.bmj.com/site/about/guidelines.xhtml

Table 1: Details of studies included in the review

	Bainey et al (2015)[18]	Rosenstock (2008)[17]	Wolak (2013)[16]	Coca (2013)[19]	Goksuluk (2015)[21]*	Weisbord (2008)[20]
**Study design**	RCT	RCT	RCT	Prospective cohort	Prospective cohort	Prospective cohort
**Sample size**	208	220	94	1017	80	44
**Country**	Canada	United States	Israel	North America	Turkey	United States
**Population**	Coronary angiography	Coronary angiography	Coronary angiography	Cardiac surgery	Coronary angiography	Coronary angiography
**Risk group**	CKD	CKD	None	High risk of AKI	Diabetes	CKD
**Mean Age (sd)**	Intervention: 73 (9) Control: 72 (8)	Intervention: 72(10) Control: 72 (10)	65(12)	Intervention: 71(11) Control: 70 (12)	NR	NR
**Female (%)**	26	52	33	31	NR	NR
**AKI definition**	Increase in SCr ≥25% or ≥0.5mg from baseline	Increase in SCr >25% or 0.5mg from baseline	Increase in SCr ≥25% from baseline	Increase in SCr ≥50% or ≥0.3mg from baseline	Increase in SCr ≥25% or ≥0.5mg from baseline	Increase in SCr ≥25% from baseline or ≥0.5mg from baseline
**Comorbidities**	Diabetes (54%), hypertension (47%), congestive heart failure (14%), liver cirrhosis (1%)	Hypotension (97%), diabetes (55%)	Diabetes (50%), unstable angina (62%)	Diabetes (47%), Hypertension (88%), congestive heart failure (23%)	Diabetes (100%)	NR
**Study drug**	ACE/ARB	ACE/ARB	ACE/ARB	ACE/ARB	ACE/ARB	NSAIDs
**Intervention: Timing of hold**	24 hours prior to procedure	Day of procedure	24 hours prior to procedure	Morning of surgery	24hrs before procedure	No details
**Intervention: timing of restart**	Up to 96 hours post procedure	24hrs post procedure	(1) Immediately afterwards; (2) 24 hours after	No details	No details	No details
**Control**	Continued throughout study	Continued throughout study	Continued throughout study	Continued throughout study	Continued throughout study	Continued throughout study
**Risk of Bias**	**Low**	**Some**: randomized by coin toss, no information on allocation concealment. Baseline difference compatable with chance	**Some**; no information on treatment allocation, baseline difference compatable with chance	**Moderate**; controlled for confounding but possibility of residual confounding	**Critical**; no control for confounding	**Not assessed**

* **Available only as CONFERENCE ABSTRACT;** RCT=randomized controlled trial; SCr=Serum creatinine; CKD=chronic kidney disease; AKI =acute kidney infection; ACE= Angiotensin-converting enzyme inhibitors,; ARB= Angiotensin receptor blockers NSAIDs= non-steroidal anti-inflammatory drugs

For peer review only - http://bmjopen.bmj.com/site/about/guidelines.xhtml

Table 2: Summary of outcomes evaluated in single studies

Outcome	Study	Effect Size (95% CI)
Urea (24 hour)	Wolak (2013)[16]	MD=2.17 [-5.22, 9.56]
Diastolic blood pressure (48 hour)	Wolak (2013)[16]	MD=0.30 [-5.01, 5.61]
Systolic blood pressure (48 hour)	Wolak (2013)[16]	MD=-2.10 [-12.98, 8.78]
Hypertensive treatment	Wolak (2013)[16]	RR=0.17 [0.01, 3.69]
Death	Bainey (2015)[18]	RR=3.15 [0.13, 78.17]
Myocardial infarction	Bainey (2015)[18]	No events
Stroke	Bainey (2015)[18]	RR=3.15 [0.13, 78.17]
Congestive heart failure	Bainey (2015)[18]	No events
Rehospitalisation	Bainey (2015)[18]	RR=7.49 [0.38, 146.89]
Interleukin 18 (IL 18) (≥120 ng/mL)	Coca (2013)[19]	0.89 [0.65, 1.23]*
Kidney injury molecule 1 (KIM 1) (≥1.15 ng/mL)	Coca (2013)[19]	1.09 [0.82, 1.44]*
Liver fatty acid binding protein (L-FABP) (≥170 ng/mL)	Coca (2013)[19]	0.97 [0.73, 1.3]*
Neutrophilgelatinase-associated lipocalin (NGAL) (≥120 ng/mL)	Coca (2013)[19]	0.84 [0.60, 1.16]*

* Adjusted for sex, age, white, CKD-EPI eGFR, diabetes, hypertension, congestive heart failure, myocardial infarction, cardiac cauterization in past 48h, electic surgery and type of surgery (CABG, valve, both)

MD=mean difference; RR=Relative risk

For peer review only

Table 3: GRADE Evidence Profile: Risks and benefits of temporarily discontinuing medications to prevent acute kidney injury

Quality assessment						№ of patients		Effect		Quality
**Studies**	**Risk of bias**	**Inconsistency**	**Indirectness**	**Imprecision**	**Other considerations**	**Continuation**	**Discontinuation**	**Relative (95% CI)**	**Absolute (95% CI)**	
**Incidence of acute kidney injury**										
3 RCTs	not serious	not serious	not serious	serious [1]	publication bias strongly suspected [2]	27/248 (10.9%)	21/274 (7.7%)	RR 1.48 (0.84 to 2.60)	52 more per 1,000 (from 17 fewer to 174 more)	⊕⊕◯◯ LOW
3 RCTs<br>3 Cohorts	very serious [3]	not serious	not serious	not serious	publication bias strongly suspected [2]	134/520 (25.8%)	323/1099 (29.4%)	RR 1.14 (0.96 to 1.36)	36 more per 1,000 (from 10 fewer to 93 more)	⊕◯◯◯ VERY LOW

CI: Confidence interval; **RR:** Risk ratio

1. Wide CI and few events
2. Non randomised studies appear would have been unlikely to have been written up for publication if findings had been negative therefore similar studies with negative findings considered likely
3. 3 RCTS, no serious concerns regarding risk of bias. 2 cohort studies, 1 judged moderate risk of bias due to possibility of residual confound, 1 judged critical risk of bias as did not control for confounding

For peer review only - http://bmjopen.bmj.com/site/about/guidelines.xhtml

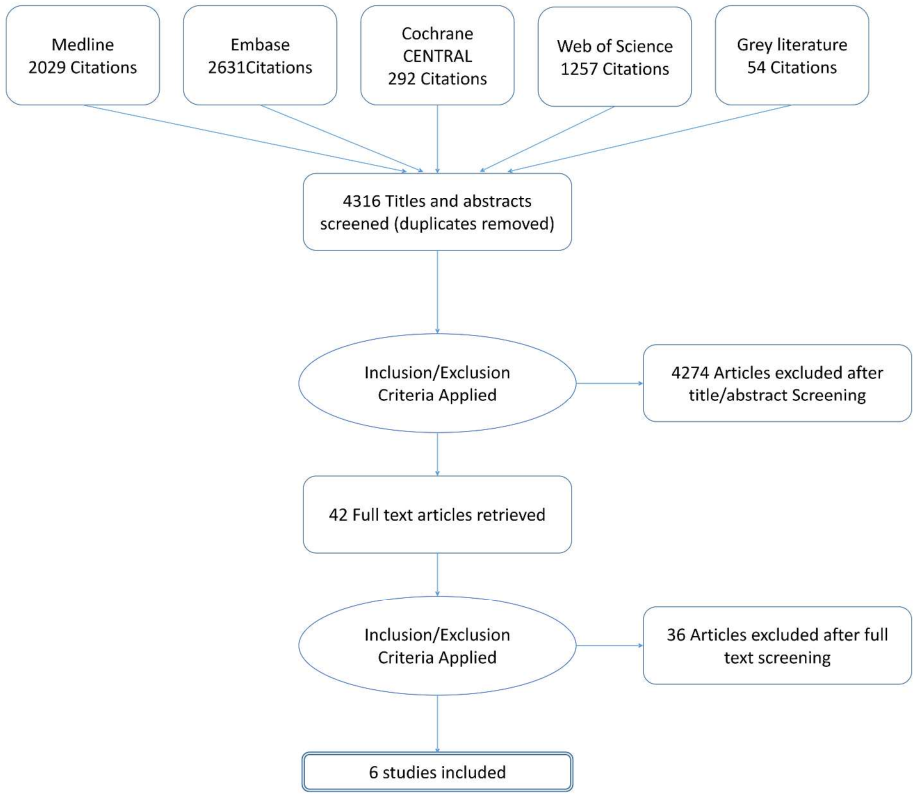

Figure 1 Flow of studies through the review process

450x451mm (300 x 300 DPI)

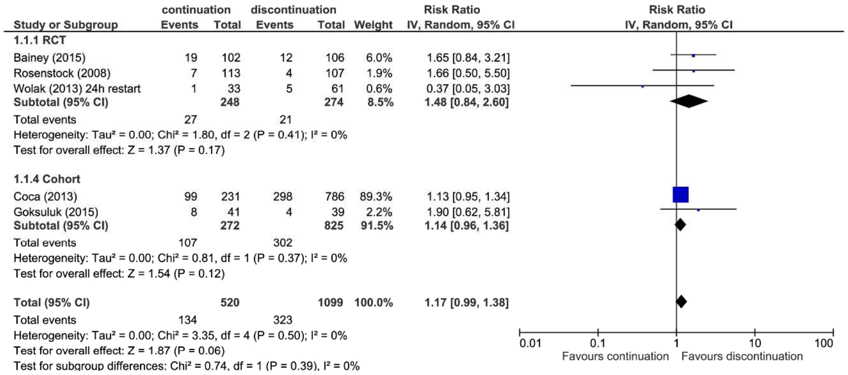

Figure 2: Forest plot showing risk of AKI in those who stopped medication prior to procedure compared to those who continued medication

215x279mm (300 x 300 DPI)

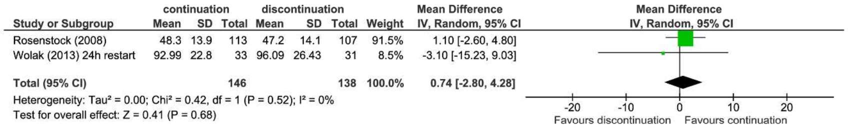

Figure 3: Forest plot showing mean difference in GFR at 24 hours in those who stopped medication prior to procedure compared to those who continued medication

215x279mm (300 x 300 DPI)

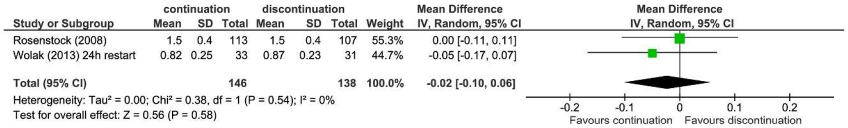

Figure 4: Forest plot showing mean difference in creatinine at 24 hours in those who stopped medication prior to procedure compared to those who continued medication

215x279mm (300 x 300 DPI)

Appendix 1: Search Strategy Medline

Language: all

Date parameters: all

Search Strategy:

1 ((sick day$ or well day$) adj2 (management or protocol$ or recommendation$ or rule$)).ti,ab. (40)

2 ((drug$ or pill$ or medicin$ or medication$) adj2 holiday$).ti,ab. (396)

3 1 or 2 (436)

4 exp "Angiotensin Receptor Antagonists"/ (17888)

5 ((angiotensin adj3 (receptor$ adj2 (antagonist$ or blocker$))) or arb or arbs).ti,ab. (11806)

6 (candesartan or amias or eprosartan or teveten or irbesartan or aprovel or coaprovel or losartan or cozaar or cozaar-comp or olmesartan or olmetec or sevikar or telmisartan or micardis or valsartan or diovan or co-diovan).ti,ab. (14149)

7 exp Angiotensin-Converting Enzyme Inhibitors/ (39220)

8 ((ace or acei or ((angiotensin adj converting adj2 enzyme*) or ace or kininase)) adj2 (inhibitor* or antagonist*)).ti,ab. (29275)

9 (captopril or ecopace or kaplon or capoten or co-zidocapt or capto-co or capozide or cilazapril or vascace or enalapril or ednyt or innovace or innozide or fosinopril or imidapril or tanatril or lisinopril or zestril or carace or zestoretic or moexipril or perdix or perindopril or coversyl or quinapril or quinil or accupro or accuretic or ramipril or tritace or triapin or trandolapril or gopten or tarka).ti,ab. (22409)

10 (renin adj4 (antagonist$ or blocker$ or inhibitor$)).ti,ab. (3552)

11 aliskiren.ti,ab. (868)

12 exp Diuretics/ (71825)

13 (diuretic$ or thiazide$ or indapamide or chlortalidone or bedroflumethiazide or xipamide or metaolozone or cyclopenthiazide or furosemide or bumetanide or torasemide or amiloride or triamterene or spironalactone or eplerenone or co-amilofruse or co-amilozide or mannitol).ti,ab. (67540)

14 exp Mineralocorticoid Receptor Antagonists/ or aldosterone antagonist$.ti,ab. (8409)

15 exp Anti-Inflammatory Agents, Non-Steroidal/ (161061)

16 (nsaid$ or ibuprofen or naproxen or fenoprofen or ketoprofen or diclofenac or aceclofenac or etodolac or indometacin or mefenamic acid or meloxicam or nabumetone or phenylbutazone or piroxicam or sulindac or tenoxicam or tolenamic acid or etoricoxib or celecoxib or acemetacin or dexibuprofen or dexketoprofen or flurbiprofen or tiaprofenic acid).ti,ab. (50654)

17 aspirin.ti,ab. (38487)

18 Metformin/ (7874)

19 metformin.ti,ab. (10748)

20 exp Sulfonylurea Compounds/ (16449)

21 (sulfonylurea$ or sulphonylurea$ or acetohexamide or carbutamide or chlorpropamide or gliclazide or glipizide or glyburide or tolazamide or tolbutamide or glibenclamide or glimepiride or glibornuride or gliquidone or glisoxepide or glyclopyramide or glimipramide).ti,ab. (20419)

22 or/4-21 (378495)

23 Withholding Treatment/ (9466)

24 (withhold$ or withheld$ or "non use" or withdraw$ or avoid$ or restart$ or continu$ or discontinu$ or stop$ or suspend$ or suspension or ceas$ or cessation).ti,ab. (1375429)

25 or/23-24 (1381746)

26 exp angiotensin ii type 1 receptor blockers/ad, ae, ct, tu, to or angiotensin ii type 2 receptor blockers/ad, ae, tu (6817)

27 exp Angiotensin-Converting Enzyme Inhibitors/ad, ae, ct, tu, to (27160)

28 "Angiotensin Receptor Antagonists"/ad, ae, ct, tu (1189)

29 exp Mineralocorticoid Receptor Antagonists/ad, ae, ct, tu, to (4429)

30 exp Diuretics/ad, ae, ct, tu, th, to or exp Anti-Inflammatory Agents, Non-Steroidal/ad, ae, ct, tu, to or Metformin/ad, ae, ct, tu, to (132402)

31 exp Sulfonylurea Compounds/ad, ae, ct, tu, to (7005)

32 ((ace$ or arb$ or diuretic$ or thiazide$ or nsaid$ or metformin or sulfonylurea$ or sulphonylurea$ or DANS) adj3 (side effect$ or adverse effect$ or adverse event$ or danger$ or injur$ or toxic$ or nephrotoxic$)).ti,ab. (5219)

For peer review only

33 or/26-32 (167054)
34 sepsis/ or exp bacteremia/ or shock, septic/ (81961)
35 (sepsis or septic).ti,ab. (94667)
36 ((toxic or endotoxic) adj shock*).ti,ab. (5911)
37 septic?emi*.ti,ab. (17808)
38 (blood stream adj2 infect*).ti,ab. (794)
39 Diarrhea/ (39641)
40 (diarrhoea* or diarrhea*).ti,ab. (81268)
41 Vomiting/ (19797)
42 (vomit* or emesis).ti,ab. (55475)
43 ((critical or serious or acute or intercurrent or concurrent) adj3 illness$).ti,ab. (20530)
44 Influenza, Human/ or *critical illness/ (47114)
45 influenza.ti,ab. (71591)
46 ((sodium or volume) adj3 depletion).ti,ab. (2605)
47 Dehydration/ (10728)
48 dehydration.ti,ab. (22570)
49 ((urinary or respiratory or skin or viral or bacterial or major or serious) adj4 infection$).ti,ab. (170172)
50 (UTI$ or RTI$).ti,ab. (534252)
51 exp *Surgical Procedures, Operative/ (1498735)
52 (surger$ or surgical or operation or operations or operativ$).ti,ab. (1585555)
53 exp Contrast Media/ (95342)
54 ((contrast$ or radiocontrast$) adj3 (agent$ or material$ or medium or media)).ti,ab. (49004)
55 or/34-54 (3590845)
56 exp Acute Kidney Injury/ (35200)
57 ((acute or early) adj (kidney or renal) adj (failure* or injur* or insufficien* or dysfunction* or impair*)).ti,ab. (31135)
58 (acute adj3 (kidney necrosis or tubul* necrosis)).ti,ab. (2972)
59 AKI.ti,ab. (4409)
60 Kidney Diseases/ci [Chemically Induced] (9718)
61 Renal Insufficiency/ci [Chemically Induced] (1216)
62 or/56-61 (58312)
63 exp *acute kidney injury/pc, ci, co, th (10689)
64 *Kidney Diseases/pc, ci, co (11347)
65 *Renal Insufficiency/pc, ci, co (1991)
66 ((AKI or acute kidney injury or acute renal injury or acute kidney failure or acute renal failure or acute kidney necrosis or acute tubular necrosis or acute kidney tubular necrosis or acute kidney insufficienc$ or acute renal insufficienc$ or acute kidney impair$ or acute renal impair$ or acute kidney dysfunction or acute renal dysfunction) adj3 (adverse event$ or adverse effect$ or mortality or morbidity or death$ or prevent$ or treat or treatment or incidence or caus$ or complication$ or minimiz$)).ti,ab. (6112)
67 or/63-66 (28252)
68 (kidney$ or renal or nephro$).mp. (929261)
69 3 and 68 (20)
70 22 and 25 and 62 (924)
71 22 and 55 and 62 (1468)
72 33 and 67 (2411)
73 69 or 70 or 71 or 72 (3765)
74 letter/ (877999)
75 editorial/ (377501)
76 news/ (168594)
77 exp historical article/ (333817)
78 Anecdotes as topic/ (4624)
79 comment/ (625733)
80 case report/ (1731454)
81 (letter or comment$).ti. (102853)
82 animals/ not humans/ (3943670)

For peer review only

83 exp Animals, Laboratory/ (745711)
84 exp Animal Experimentation/ (6628)
85 exp Models, Animal/ (436993)
86 exp rodentia/ (2737281)
87 (rat or rats or mouse or mice).ti. (1142491)
88 or/74-87 (8050101)
89 73 not 88 (2085)
90 exp child/ or exp infant/ or (child$ or infant$ or neonat$ or newborn$ or baby or babies or p?ediatric).ti. (2239441)
91 exp adult/ or adult$.ti. (5842639)
92 90 not 91 (1539523)
93 89 not 92 (1924)

For peer review only

Section/topic	#	Checklist item	Reported on page #
**TITLE**			
Title	1	Identify the report as a systematic review, meta-analysis, or both.	1
**ABSTRACT**			
Structured summary	2	Provide a structured summary including, as applicable: background; objectives; data sources; study eligibility criteria, participants, and interventions; study appraisal and synthesis methods; results; limitations; conclusions and implications of key findings; systematic review registration number.	2
**INTRODUCTION**			
Rationale	3	Describe the rationale for the review in the context of what is already known.	4
Objectives	4	Provide an explicit statement of questions being addressed with reference to participants, interventions, comparisons, outcomes, and study design (PICOS).	4
**METHODS**			
Protocol and registration	5	Indicate if a review protocol exists, if and where it can be accessed (e.g., Web address), and, if available, provide registration information including registration number.	5
Eligibility criteria	6	Specify study characteristics (e.g., PICOS, length of follow-up) and report characteristics (e.g., years considered, language, publication status) used as criteria for eligibility, giving rationale.	5
Information sources	7	Describe all information sources (e.g., databases with dates of coverage, contact with study authors to identify additional studies) in the search and date last searched.	5
Search	8	Present full electronic search strategy for at least one database, including any limits used, such that it could be repeated.	Web Appendix
Study selection	9	State the process for selecting studies (i.e., screening, eligibility, included in systematic review, and, if applicable, included in the meta-analysis).	6

For peer review only

Section/topic	#	Checklist item	Reported on page #
Data collection process	10	Describe method of data extraction from reports (e.g., piloted forms, independently, in duplicate) and any processes for obtaining and confirming data from investigators.	6
Data items	11	List and define all variables for which data were sought (e.g., PICOS, funding sources) and any assumptions and simplifications made.	6
Risk of bias in individual studies	12	Describe methods used for assessing risk of bias of individual studies (including specification of whether this was done at the study or outcome level), and how this information is to be used in any data synthesis.	6
Summary measures	13	State the principal summary measures (e.g., risk ratio, difference in means).	7
Synthesis of results	14	Describe the methods of handling data and combining results of studies, if done, including measures of consistency (e.g., I^2) for each meta-analysis.	7
Risk of bias across studies	15	Specify any assessment of risk of bias that may affect the cumulative evidence (e.g., publication bias, selective reporting within studies).	7
Additional analyses	16	Describe methods of additional analyses (e.g., sensitivity or subgroup analyses, meta-regression), if done, indicating which were pre-specified.	7
**RESULTS**			
Study selection	17	Give numbers of studies screened, assessed for eligibility, and included in the review, with reasons for exclusions at each stage, ideally with a flow diagram.	7; Figure 1
Study characteristics	18	For each study, present characteristics for which data were extracted (e.g., study size, PICOS, follow-up period) and provide the citations.	8; Table 1
Risk of bias within studies	19	Present data on risk of bias of each study and, if available, any outcome level assessment (see item 12).	8; Table 1

For peer review only

Section/topic	#	Checklist item	Reported on page #
Results of individual studies	20	For all outcomes considered (benefits or harms), present, for each study: (a) simple summary data for each intervention group (b) effect estimates and confidence intervals, ideally with a forest plot.	Figure 2
Synthesis of results	21	Present results of each meta-analysis done, including confidence intervals and measures of consistency.	Figure 2; Table 2
Risk of bias across studies	22	Present results of any assessment of risk of bias across studies (see Item 15).	Table 3
Additional analysis	23	Give results of additional analyses, if done (e.g., sensitivity or subgroup analyses, meta-regression [see Item 16]).	Figure 2
**DISCUSSION**			
Summary of evidence	24	Summarize the main findings including the strength of evidence for each main outcome; consider their relevance to key groups (e.g., healthcare providers, users, and policy makers).	Table 3; 11
Limitations	25	Discuss limitations at study and outcome level (e.g., risk of bias), and at review-level (e.g., incomplete retrieval of identified research, reporting bias).	12
Conclusions	26	Provide a general interpretation of the results in the context of other evidence, and implications for future research.	15
**FUNDING**			
Funding	27	Describe sources of funding for the systematic review and other support (e.g., supply of data); role of funders for the systematic review.	16

For peer review only
